# Piggybacking on Niche Adaptation Improves the Maintenance of Multidrug‐Resistance Plasmids

**DOI:** 10.1093/molbev/msab091

**Published:** 2021-03-24

**Authors:** Julia Kloos, João A Gama, Joachim Hegstad, Ørjan Samuelsen, Pål J Johnsen

**Affiliations:** 1 Department of Pharmacy, Faculty of Health Sciences, UiT The Arctic University of Norway, Tromsø, Norway; 2 Department of Microbiology and Infection Control, University Hospital of North Norway, Tromsø, Norway; 3 Norwegian National Advisory Unit on Detection of Antimicrobial Resistance, Department of Microbiology and Infection Control, University Hospital of North Norway, Tromsø, Norway

**Keywords:** plasmids, fitness cost, antibiotic resistance, clinical isolates, experimental evolution, niche adaptation

## Abstract

The persistence of plasmids in bacterial populations represents a puzzling evolutionary problem with serious clinical implications due to their role in the ongoing antibiotic resistance crisis. Recently, major advancements have been made toward resolving this “plasmid paradox” but mainly in a nonclinical context. Here, we propose an additional explanation for the maintenance of multidrug‐resistance plasmids in clinical *Escherichia coli* strains. After coevolving two multidrug‐resistance plasmids encoding resistance to last resort carbapenems with an extraintestinal pathogenic *E. coli* strain, we observed that chromosomal media adaptive mutations in the global regulatory systems CCR (carbon catabolite repression) and ArcAB (aerobic respiration control) pleiotropically improved the maintenance of both plasmids. Mechanistically, a net downregulation of plasmid gene expression reduced the fitness cost. Our results suggest that global chromosomal transcriptional rewiring during bacterial niche adaptation may facilitate plasmid maintenance.

## Introduction

Plasmids are self-replicating extrachromosomal elements that often decrease bacterial fitness due to the requirement of host functions for their own replication and spread (reviewed in Baltrus 2013; San Millan and MacLean 2017), although beneficial (or noncostly) plasmids have been reported (Enne et al. 2004; Monarrez et al. 2019). These genetic elements play a key role in the evolution and spread of antibiotic resistance determinants in bacterial populations world-wide (Carattoli 2013; Partridge et al. 2018). This is particularly true for nosocomial pathogens in the family *Enterobacteriaceae* including *Escherichia coli* and *Klebsiella pneumoniae* where resistance determinants of high clinical relevance such as carbapenemases and extended-spectrum β-lactamases are frequently encoded on plasmids (Mathers et al. 2015; Rozwandowicz et al. 2018).

From an evolutionary perspective, persistence of plasmids in bacterial populations has for a long time been a conundrum often referred to as the “plasmid paradox” (Stewart and Levin 1977; Harrison and Brockhurst 2012). This paradox can be resolved in at least five different ways. First, maintenance can be ensured by positive selection for plasmid-encoded traits (Gullberg et al. 2014; San Millan et al. 2014; Stevenson et al. 2018). But, if too beneficial, positively selected traits may be captured by the chromosome rendering the plasmid obsolete and consequently lost, as demonstrated theoretically (Bergstrom et al. 2000) and experimentally (Kottara et al. 2018). Second, mathematical models predict that high rates of horizontal plasmid transfer can counteract segregational plasmid loss and the competitive disadvantage of plasmid-carriers (Stewart and Levin 1977). *In vitro* studies report that conditions exist where conjugation frequencies are indeed extremely high (Dionisio et al. 2002; Lopatkin et al. 2017). It is however generally accepted that conjugation is a costly process (San Millan and MacLean 2017) and evolution toward increased conjugation rates does not constitute a general solution of the paradox (Turner et al. 1998; Dahlberg and Chao 2003; Porse et al. 2016). Third, transmissible plasmids under purifying selection may “escape” their host and enter less hostile environments. This has been termed cross-ecotype transfer (Bergstrom et al. 2000). Fourth, plasmid stability can evolve through improved replication control (Wein et al. 2019) and the acquisition of addiction mechanisms (Loftie-Eaton et al. 2016). Fifth, and perhaps most prominent, negative effects on host fitness can be mitigated through compensatory evolution (San Millan and MacLean 2017), and plasmids may even become beneficial (Bouma and Lenski 1988; Dionisio et al. 2005; Starikova et al. 2013; Loftie-Eaton et al. 2017). Fitness compensating mutations have been demonstrated to occur both in the presence and absence of selective agents and were identified on bacterial chromosomes (San Millan et al. 2014; Harrison et al. 2015; Loftie-Eaton et al. 2017), on plasmids (De Gelder et al. 2008; Sota et al. 2010; Porse et al. 2016), or both (Dahlberg and Chao 2003; Starikova et al. 2013; Bottery et al. 2017).

The last 10 years have brought significant advancements in the understanding of plasmid–host evolutionary dynamics. However, it is not clear how the different solutions to the plasmid paradox as listed above are relevant for clinical strains and plasmids since the majority of published work has focused on emblematic laboratory strains and/or environmental bacteria. In this report, we asked if and how two clinical plasmids encoding the VIM-1 and NDM-1 carbapenemases affected fitness of an *E. coli* strain isolated from a patient, before and after experimental evolution. We observed striking parallel evolution of the CCR (carbon catabolite repression) and ArcAB (aerobic respiration control) regulatory systems in the chromosomes of both plasmid-containing and plasmid-free populations resulting in adaptation to the experimental conditions. No apparent plasmid-specific compensatory mutations were identified across evolved populations and the plasmid sequences were largely unchanged. Yet, the initial plasmid costs were ameliorated in the coevolved cultures. We demonstrate that fitness amelioration resulted from “piggybacking” on the clinical strains’ adaptation to a new niche, suggesting a novel solution to the “plasmid paradox.”

## Results

### Plasmid Acquisition Moderately Reduces Fitness in a Clinical *E. coli* Host Strain

**Fig. 1.**
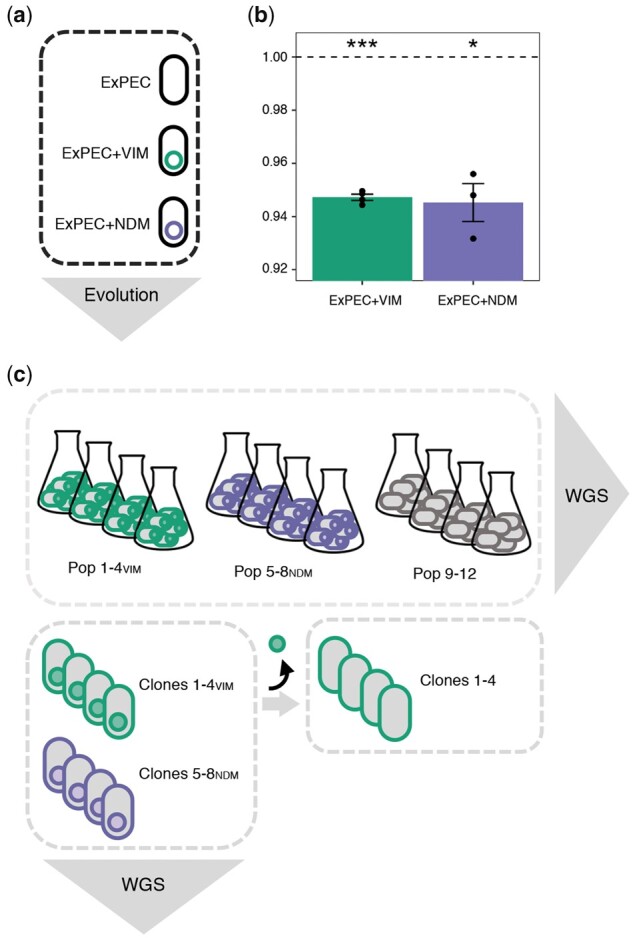
Fitness effect of plasmid acquisition and experimental procedures. (*a*) An ExPEC strain (black) acquired each of the two MDR plasmids pG06-VIM-1 (green; 53 kB; IncR; Di Luca et al. 2017) and pK71-77-1-NDM (purple; 145 kB; IncC; Gama et al. 2020) of clinical origin encoding the carbapenemases VIM-1 and NDM-1, respectively. (*b*) Initial fitness costs of newly transferred plasmids in strains ExPEC+VIM and ExPEC+NDM (*n* = 4 and 3, respectively). Significant plasmid costs are indicated by asterisks (*P *=* ** < 0.05, ** < 0.01, *** < 0.001; one-sample *t*-test, two-sided). Error bars indicate ±SEM. (*c*) Experimental evolution in absence of selective pressure (∼300 generations) resulted in plasmid-carrying (Pop 1–4_VIM_ and Pop 5–8_NDM_) and plasmid-free (Pop 9–12) populations which were subjected to whole-genome sequencing (WGS). Representative clones per plasmid-carrying evolved population (Clones 1–4_VIM_ and Clones 5–8_NDM_) were sequenced and segregants without evolved pG06-VIM-1 (filled green circle) were generated for subsequent competition experiments (Clones 1–4).

To mimic the acquisition of plasmid-mediated resistance to a last resort antibiotic, we transferred each of the two carbapenemase-producing clinical plasmids pG06-VIM-1 from *K. pneumoniae* (*bla*_VIM-1_; Samuelsen et al. 2011) and pK71-77-1-NDM from *E. coli* (*bla*_NDM-1_; Samuelsen et al. 2011) into an extraintestinal pathogenic *E. coli* sequence type (ST) 537 (strain ExPEC; Bengtsson et al. 2012; supplementary table 1, Supplementary Material online). pG06-VIM-1 is nonconjugative (Di Luca et al. 2017), whereas pK71-77-1-NDM is conjugative (Gama et al. 2020). Plasmid transfer resulted in strains ExPEC+VIM and ExPEC+NDM, both otherwise isogenic to strain ExPEC (fig. 1*a* and supplementary table 1, Supplementary Material online).

We measured the cost of the newly introduced clinical plasmids in head-to-head competition experiments lasting approximately 40 generations. Acquisition of either pG06-VIM-1 or pK71-77-1-NDM affected host fitness similarly, resulting in moderate but significant costs of 5.3% and 5.5%, respectively (one-sample *t*-test, two-sided: ExPEC+VIM: *w *=* *0.947 ± 0.002, *P *<* *0.001; ExPEC+NDM: *w *=* *0.945 ± 0.012, *P *=* *0.017; fig. 1*b* and supplementary table 6, Supplementary Material online).

### Strong Parallel Evolution in Global *E. coli* Regulators Occurs Independently of Plasmid-Carriage

Four replicate lineages of the plasmid-containing strains ExPEC+VIM and ExPEC+NDM as well as the plasmid-free strain ExPEC were serially transferred for approximately 300 generations (over which the plasmids are stably maintained; Di Luca et al. 2017; Gama et al. 2020). This resulted in 12 evolved populations (Pop 1–4_VIM_, Pop 5–8_NDM_, and Pop 9–12; fig. 1*c* and supplementary table 1, Supplementary Material online), that we deep sequenced to identify putative mutations mitigating the fitness costs of plasmid carriage.

**Fig. 2.**
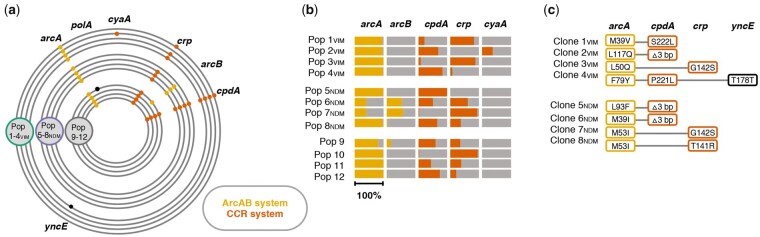
Identified mutations in the ArcAB (aerobic respiration control) and CCR (carbon catabolite repression) regulatory systems. (*a*) Chromosomal mutations after approximately 300 generations of experimental evolution. Plasmid-carrying (Pop 1–4_VIM_* *=* *green; Pop 5–8_NDM_* *=* *purple) and plasmid-free (Pop 9–12 = gray) populations had acquired chromosomal mutations in genes associated to the ArcAB (yellow) and CCR (orange) regulatory system. Black indicates otherwise mutated genes in single evolved populations. No point mutations were identified in plasmid sequences. See also supplementary section IIIa and supplementary table 4, Supplementary Material online. (*b*) Total frequency of all mutations targeting the same gene within single evolved populations for genes linked to ArcAB and CCR regulatory systems (entire bar length* *=* *100%). (*c*) Chromosomal mutations identified in coevolved, plasmid-carrying, whole-genome sequenced Clones 1–4_VIM_ and Clones 5–8_NDM_; “Δ3 bp” in *cpdA *=* cpdA*.Δ3.bp488-490.

At the population level, no changes were identified in the evolved plasmid sequences except in Pop 5_NDM_ harboring a deletion in the evolved pK71-77-1-NDM (supplementary section IIIa, supplementary table 3, and supplementary fig. 1, Supplementary Material online). However, all 12 evolved lineages revealed patterns of extensive parallel evolution in chromosomal genes that are directly or indirectly linked to the CCR and the ArcAB regulatory systems of *E. coli* (fig. 2*a*)*.* In total, 68 different mutations were identified in genes *cpdA* (3′,5′-cyclic adenosine monophosphate [cAMP] phosphodiesterase), *crp* (cAMP receptor protein; DNA-binding transcriptional regulator), *arcA* (aerobic respiration control protein; DNA-binding transcriptional regulator), and *arcB* (aerobic respiration control sensor protein; histidine kinase). Evolved lineages had on average acquired eight variations in these genes ranging from three (Pop 5_NDM_) to 18 (Pop 6_NDM_) different mutations for individual populations (supplementary fig. 2, Supplementary Material online). Our data revealed 25, 12, 23, and 8 unique mutations in *arcA* (717 bp)*, arcB* (2,337 bp)*, cpdA* (828 bp), and *crp* (633 bp), respectively (supplementary fig. 2, Supplementary Material online)*.* Among these unique mutations in the respective target genes, 12, one, three, and three were found repeatedly across more than one evolved population. The majority of mutations in these genes were nonsynonymous single nucleotide exchanges leading to amino acid substitutions (88%). Furthermore, Pop 2_VIM_ acquired mutations upstream and in the open-reading frame of *cyaA* (adenylate cyclase; cAMP synthesis). For a detailed list of mutations identified across evolved populations, including small indels, as well as mutations found in single populations, see supplementary table 4, Supplementary Material online. Whereas *cpdA* and *arcA* were mutation targets in all 12 populations, *crp* and *arcB* were identified in ten and four populations, respectively (fig. 2*a* and supplementary fig. 2, Supplementary Material online). Surprisingly, the mutation profiles were not different in populations that coevolved with any of the plasmids compared with the plasmid-free control populations, strongly suggesting that the observed mutational changes were not plasmid-specific. Genes of the CCR and ArcAB systems are indeed frequently reported as mutational targets for adaptive responses to the experimental growth conditions occurring during laboratory evolution experiments (Knoppel et al. 2018; Phaneuf et al. 2019).

### Mutations in CCR and ArcAB Regulatory Systems Pleiotropically Mitigate the Cost of pG06-VIM-1 and pK71-77-1-NDM Carriage

Immediate acquisitions of pG06-VIM-1 and pK71-77-1-NDM reduced host fitness significantly (fig. 1*b*). We have previously demonstrated complete retention of the same plasmids following experimental evolution under the same antibiotic-free conditions (Di Luca et al. 2017; Gama et al. 2020). Since one of the plasmids was nonconjugative, we assumed that fitness amelioration by compensatory adaptation was the most likely route for the plasmids to persist in evolved populations. However, the sequencing data presented above revealed no apparent plasmid-specific compensatory mutations. Therefore, we hypothesized that adaptation to the growth conditions could have pleiotropic effects on the costs of plasmid carriage.

To test this hypothesis, we isolated a single clone from each evolved plasmid-carrying population (Pop 1–4_VIM_ and Pop 5–8_NDM_) with mutations in both regulatory systems, CCR and ArcAB, since population sequencing data suggested that both systems were affected simultaneously (fig. 2*a* and *b*). In the selected Clones 1–4_VIM_ and Clones 5–8_NDM_, Sanger and Illumina sequencing confirmed the presence of mutations as expected from population sequencing results and no further chromosomal or plasmid-located point mutations (figs. 1*c* and 2*c*; supplementary sections I and IIIb and supplementary table 1, Supplementary Material online).

Here, we also identified large deletions in the evolved pK71-77-1-NDM for Clone 5_NDM_ and Clone 7_NDM_ (∼8.8 and ∼58.9 kb, respectively; supplementary section IIIb, supplementary table 3, and supplementary fig. 1, Supplementary Material online), and susceptibility testing by disc diffusion phenotypically confirmed the deletions involving antibiotic resistance genes (supplementary section VI and supplementary table 8, Supplementary Material online). The plasmid copy-number for pG06-VIM-1 and pK71-77-1-NDM before and after experimental evolution was unchanged (0.9–1.5 copies in all sequenced plasmid-carrying clones based on read coverage; supplementary section IIIb and supplementary table 5, Supplementary Material online). Next, we attempted to isolate a set of spontaneous plasmid-free segregants of Clones 1–4_VIM_ and Clones 5–8_NDM,_ to use in competition experiments, by screening for ampicillin-susceptible colonies. We obtained segregants for pG06-VIM-1 resulting in Clones 1–4, but not for pK71-77-1-NDM, which we confirmed by Sanger sequencing to have the niche-adaptive mutations (fig. 1*c*). Illumina sequencing of Clones 2 and 3 verified that no chromosomal mutations were acquired during the curing procedure.

**Fig. 3.**
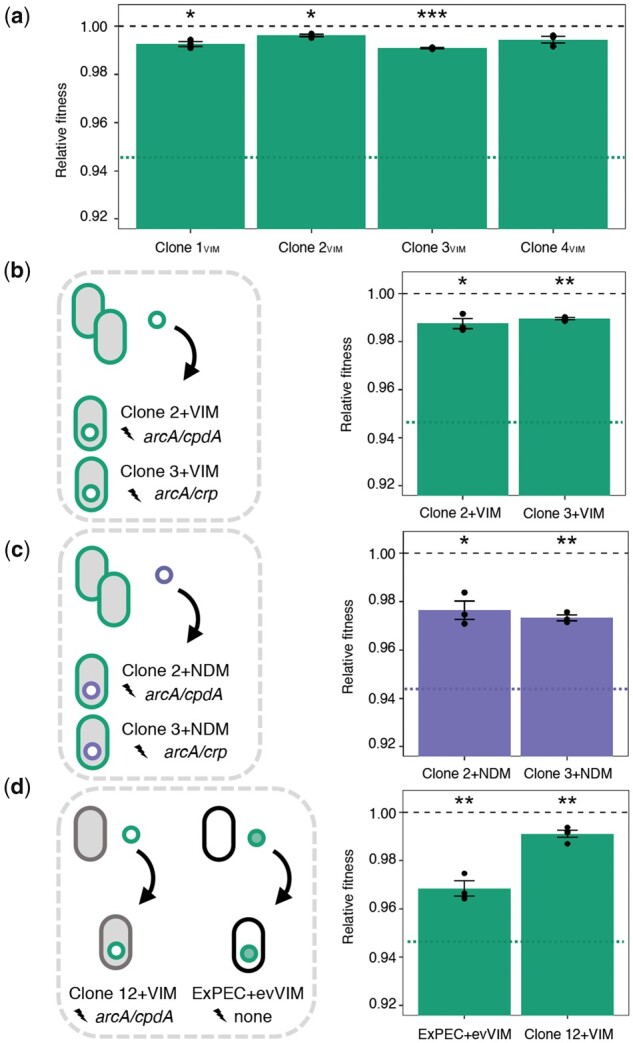
Fitness costs of evolved and ancestral plasmids in adapted backgrounds. (*a*) Relative fitness of coevolved pG06-VIM-1-carrying clones (*n* = 3). Fitness of the ancestral strain ExPEC+VIM is indicated by a dotted green line. (*b*) Fitness cost of ancestral pG06-VIM-1 reintroduced into coevolved Clone 2 and Clone 3. Fitness of ancestral strain ExPEC+VIM is indicated by a dotted green line. (*c*) Fitness cost of ancestral pK71-77-1-NDM introduced into coevolved Clone 2 and Clone 3 (*n* = 3). Fitness of the ancestral strain ExPEC+NDM is indicated by a dotted purple line. (*d*) Fitness cost of ancestral pG06-VIM-1 introduced into evolved Clone 12, and of evolved pG06-VIM-1 isolated from coevolved Clone2_VIM_ introduced into ancestral strain ExPEC (*n* = 3–4). Significant plasmid costs are indicated by asterisks (*P *=* ** < 0.05, ** < 0.01, *** < 0.001; one-sample *t*-test, two-sided). Error bars indicate ±SEM.

The costs of pG06-VIM-1-carriage in coevolved Clones 1–4_VIM_ were assessed in head-to-head competitions with the respective plasmid-free isogenic strains (Clones 1–4) over approximately 40 generations. Our data show that the initial costs were significantly ameliorated to ≤1% in all four evolved backgrounds irrespective of the combination of chromosomal mutations in these clones (one-sample *t*-test, two-sided: Clone 1_VIM_: 0.7% or *w *=* *0.993 ± 0.002, *P *=* *0.017; Clone 2_VIM_: 0.4% or *w *=* *0.996 ± 0.001, *P *=* *0.016; Clone 3_VIM_: 0.9% or *w *=* *0.991 ± 0.0003, *P *=* *0.001 and Clone 4_VIM_: 0.6% or *w *=* *0.994 ± 0.002, *P *=* *0.056; one-way ANOVA assuming equal variances, df* *=* *4, *P *<* *0.001, followed by Dunnett’s test: *P *<* *0.001; fig. 3*a* and supplementary table 6, Supplementary Material online). Illumina sequencing confirmed that the plasmid sequences in Clones 1–4_VIM_ were unchanged after evolution suggesting that the chromosomal mutations were responsible for the fitness mitigation. To further test this, we introduced the ancestral pG06-VIM-1 into Clone 2 and Clone 3 carrying mutations in *arcA/cpdA* and *arcA/crp*, respectively, resulting in Clone 2+VIM and Clone 3+VIM (fig. 3*b*). Competition experiments with the isogenic, plasmid-free genetic backgrounds revealed a significant fitness increase compared with the original plasmid–host combination and an amelioration of the initial cost of harboring pG06-VIM-1 to 1.3% and 1%, respectively (one-sample *t*-test, two-sided: Clone 2+VIM: *w *=* *0.987 ± 0.004, *P *=* *0.026; Clone 3+VIM: *w *=* *0.990 ± 0.001, *P *=* *0.002; one-way ANOVA assuming equal variances, df* *=* *2, *P *<* *0.001, followed by Dunnett’s test: *P *<* *0.001; fig. 3*b* and supplementary table 6, Supplementary Material online). To exclude that plasmid-specific adaptation in these pG06-VIM-1-coevolved clones was responsible for the observed fitness amelioration, we introduced the ancestral pG06-VIM-1 into an isolated clone of Pop 12 (Clone 12+VIM; fig. 3*d*) and determined fitness as described above. In this background, which had evolved without a plasmid and acquired mutations in *arcA/cpdA*, the cost of pG06-VIM-1-carriage was also significantly reduced to ≤1% (one-sample *t*-test, two-sided: Clone 12+VIM: 0.9% or *w *=* *0.991 ± 0.002, *P *=* *0.009, fig. 3*d*). Although this was significantly different from the initial plasmid cost, it did not differ from the cost of ancestral pG06-VIM-1 in coevolved Clones 2+VIM and 3+VIM (one-way ANOVA assuming equal variances, df* *=* *3, *P *<* *0.001, followed by Tukey’s test; supplementary table 6, Supplementary Material online).

Similarly, the initial fitness cost of 5.5% imposed by the ancestral pK71-77-1-NDM in strain ExPEC+NDM was significantly decreased in pG06-VIM-1-free segregants carrying this plasmid (one-sample *t*-test, two-sided: Clone 2+NDM: 2.4% or *w *=* *0.976 ± 0.007; *P *=* *0.025; Clone 3+NDM: 2.7% or *w *=* *0.973 ± 0.002; *P *=* *0.002; one-way ANOVA assuming equal variances, df* *=* *2, *P *=* *0.006, followed by Dunnett’s test: *P *=* *0.006 and *P *=* *0.010, respectively; fig. 3*c* and supplementary table 6, Supplementary Material online). Note that we did not obtain a plasmid-free background of Clones 5–8_NDM_ to test the fitness of pK71-77-1-NDM-coevolved strains. The results from competition experiments with Clone 2+NDM and Clone 3+NDM carrying the ancestral pK71-77-1-NDM strongly suggest that the chromosomal mutations are responsible for partial fitness amelioration. We acknowledge that the deletions in evolved plasmids of Clone 5_NDM_ and Clone 7_NDM_, removing conjugation and resistance genes, could result in further fitness improvements as demonstrated previously (Turner et al. 2014; Porse et al. 2016).

We transformed the evolved pG06-VIM-1 from Clone 2_VIM_ into the ancestral ExPEC strain (fig. 3*d*) to test for adaptive changes occurring in plasmid sequences that could be undetected using short-read sequencing. The evolved pG06-VIM-1 affected the ancestral host significantly (one-sample *t*-test, two-sided: ExPEC+evVIM: 3.2% or *w *=* *0.968 ± 0.005, *P *=* *0.01) being less costly than the ancestral pG06-VIM-1, but more costly in the ancestral than in an adapted background (one-way ANOVA assuming equal variances, df* *=* *3, *P *<* *0.001, followed by Tukey’s test; fig. 3*d* and supplementary table 6, Supplementary Material online). Some undetected plasmid mutations may reduce the cost of the evolved plasmid, however to a lesser extent than the mutations in CCR and ArcAB systems as demonstrated with Clone 12+VIM above.

Taken together, our data indicate clearly that the different mutations identified in the CCR and ArcAB regulatory systems are sufficient to improve plasmid maintenance. The usage of isogenic strains, distinguishable only by plasmid-encoded markers is however a limitation that could skew the accuracy of the fitness measurements because of plasmid loss or conjugation. Plasmid loss could lead to an overestimation of the fitness cost, but during the approximately 40 generations of fitness measurement (see Materials and Methods and supplementary section IV, Supplementary Material online) all tested plasmids were stable (supplementary table 7, Supplementary Material online) such that the effect of this parameter can be neglected. This is further corroborated by our inability to select any spontaneous pK71-77-1-NDM-free segregant. We also measured the conjugation efficiencies for pK71-77-1-NDM (12 h) in additional experiments (see Materials and Methods and supplementary section V, Supplementary Material online), revealing small but significantly increased plasmid transfer frequencies in the adapted backgrounds (supplementary fig. 3, Supplementary Material online). This effect could lead to underestimation of the fitness cost of pK71-77-1-NDM in evolved hosts. Nevertheless, we conclude that the mutations in the regulatory systems improve the maintenance of this plasmid, either directly reducing the fitness cost or through increased conjugative transfer.

### Plasmid Cost Mitigation Is Linked Specifically to the CCR System

**Fig. 4.**
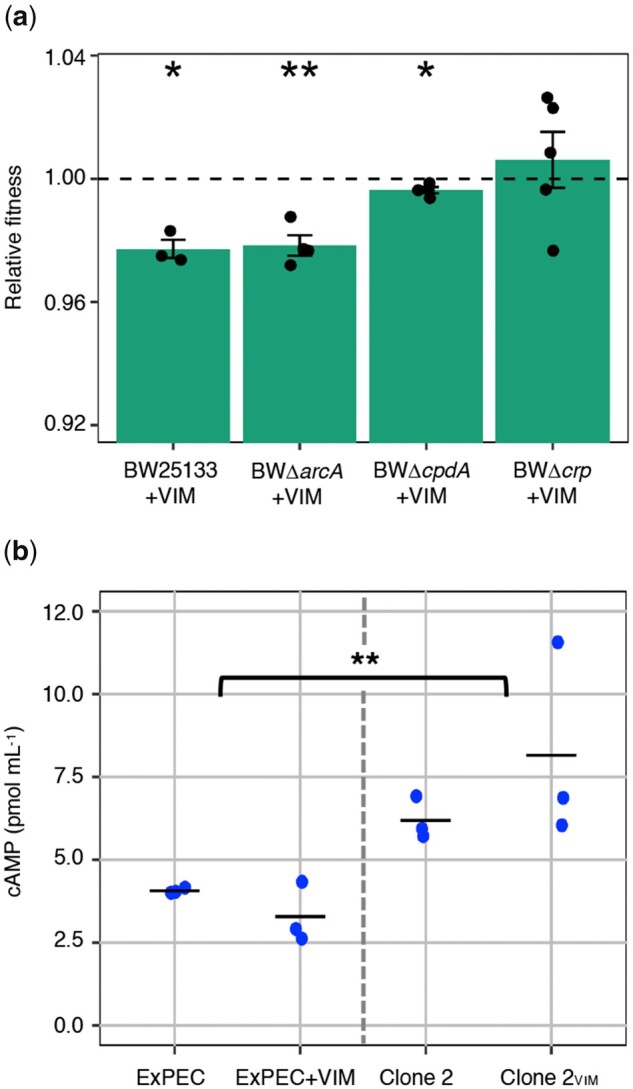
Effect of CCR and ArcAB systems mutations on plasmid cost and intracellular cAMP concentration. (*a*) Relative fitness of pG06-VIM-1 in parent strain BW25133 and deletion strains (*n* = 3–5). Significant plasmid costs are indicated by asterisks (*P *=* ** < 0.05, ** < 0.01, *** < 0.001; one-sample *t*-test, two-sided). Error bars indicate ±SEM. (*b*) Intracellular cAMP concentrations of ancestral strains (ExPEC and ExPEC+VIM; *n* = 6; left) and evolved strains (Clone 2 and Clone 2_VIM_ carrying mutation *cpdA*.Δ3.bp488-490; *n* = 6; right) (two-way ANOVA; *P *=* *** < 0.01; df* *=* *3).

To investigate the individual roles of the CCR and ArcAB systems on plasmid cost mitigation, we measured plasmid costs in deletion mutants for *arcA, cpdA*, and *crp —* the targeted loci for adaptation in our coevolved clones. Unfortunately, genetic modifications using clinical strains are notoriously difficult and for these experiments, we used deletion mutants of *E. coli* (K-12 derivatives) from the Keio collection (Baba et al. 2006). We introduced the ancestral pG06-VIM-1 into the individual deletion strains as well as the Keio parent strain (Datsenko and Wanner 2000) by electroporation, resulting in strains BW25113+VIM, BWΔ*cpdA*+VIM, BWΔ*arcA*+VIM, and BWΔ*crp*+VIM (supplementary section I and supplementary table 1, Supplementary Material online). We measured fitness of plasmid-carrying strains relative to their plasmid-free counterparts in head-to-head competitions as described above. As a general observation, pG06-VIM-1 was less costly in BW25113 than in the clinical isolate (one-sample *t*-test, two-sided: 2.3% or *w *=* *0.977 ± 0.005, *P *=* *0.016; fig. 4*a* and supplementary table 6, Supplementary Material online). Although deletion of *arcA* and *crp* had no significant effect on the fitness burden imposed by pG06-VIM-1 compared with BW25113+VIM, we measured a significant fitness improvement of the pG06-VIM-1-carrying Δ*cpdA* mutant (one-way ANOVA not assuming equal variances, df* *=* *3, *P *=* *0.004 followed by Dunnett’s test: BWΔ*arcA*+VIM, *P *=* *0.993; BWΔ*crp*+VIM, *P *=* *0.051; BWΔ*cpdA*+VIM, *P *=* *0.001; fig. 4*a* and supplementary table 6, Supplementary Material online) and a reduction of plasmid cost to 0.4% (one-sample *t*-test, two-sided: BWΔ*arcA*+VIM: 2.2% or *w *=* *0.978 ± 0.007, *P *=* *0.007; BWΔ*crp*+VIM: no cost or *w *=* *1.006 ± 0.020, *P *=* *0.534; BWΔ*cpdA*+VIM: *w *=* *0.996 ± 0.002, *P *=* *0.034; fig. 4*a* and supplementary table 6, Supplementary Material online). These data strongly suggest that beyond the known effect on adaptation to growth conditions, *cpdA* mutations identified in our study pleiotropically mitigated plasmid costs. We could however not obtain the same level of consistency across biological replicates in competitions using the Δ*crp* mutant even though CpdA and CRP are tightly linked in the CCR regulatory system (Imamura et al. 1996; Matange 2015). Based on the observation that pG06-VIM-1 no longer imposes a cost in the Δ*crp* mutant, as confirmed by the one-sample *t*-test, we argue that *crp* is also involved in fitness mitigation despite the relatively high variance that renders the Dunnett’s test (borderline) not significant.

In *E. coli*, the phosphodiesterase CpdA affects intracellular levels of cAMP by specifically hydrolyzing this signaling molecule (Imamura et al. 1996). To investigate the effect of the most frequently observed mutation in *cpdA* (*cpdA*.Δ3.bp488-490; supplementary table 4, Supplementary Material online) on protein function, we measured intracellular cAMP concentrations in ancestral and evolved strains with and without pG06-VIM-1. The levels of intracellular cAMP increased significantly by 49% between ancestral and evolved strains (ExPEC and ExPEC+VIM: 2.6 to 4.3 pmol mL^−1^, mean* *=* *3.7 ± 0.72 pmol mL^−1^; Clone 2 and Clone 2_VIM_: 5.7 to 11.6 pmol mL^−1^, mean* *=* *7.2 ± 2.2 pmol mL^−1^; two-way ANOVA with interactions: df* *=* *3, *P *=* *0.005 [assuming equal variances] and *P *=* *0.001 [adjusted for unequal variances]; fig. 4*b*), but were unaffected by plasmid presence (two-way ANOVA with interactions: df* *=* *3, *P *=* *0.53 [assuming equal variances] and *P *=* *0.40 [adjusted for unequal variances]). These data are consistent with the previously observed CpdA deficiency of an identical *E. coli* mutant resulting in an approximate doubling of intracellular cAMP (Chib and Seshasayee 2018). Similarly, protein function analysis of evolved population data indicates that the majority of mutations in *cpdA* lead to the loss of CpdA function (supplementary section VII and supplementary table 9, Supplementary Material online).

### Mutations in CCR and ArcAB Regulatory Systems Lead to General Adaptation to the Growth Conditions

The gene products of *cyaA, cpdA*, *crp*, *arcA*, *arcB* can be associated with transcription in *E. coli* involving the global regulators CRP and ArcA (Martínez-Antonio and Collado-Vides 2003). cAMP is an important second messenger that binds to CRP (Frendorf et al. 2019) and the complex activates cAMP-dependent regulation of carbon source utilization via the CCR system (Imamura et al. 1996; Matange 2015). Intracellular levels of cAMP in *E. coli* are controlled by CyaA (synthesis) and CpdA (degradation) (Imamura et al. 1996). Proteins ArcA and ArcB compose the ArcAB two-component regulatory system involved in respiratory and energy metabolism of *E. coli* (Iuchi and Lin 1988; Iuchi et al. 1989). Mutations in CCR- and ArcAB-associated proteins may lead to growth optimization in varying environments due to adaptation in downstream transcriptional regulatory networks (Saxer et al. 2014; Frendorf et al. 2019; Phaneuf et al. 2019).

**Fig. 5.**
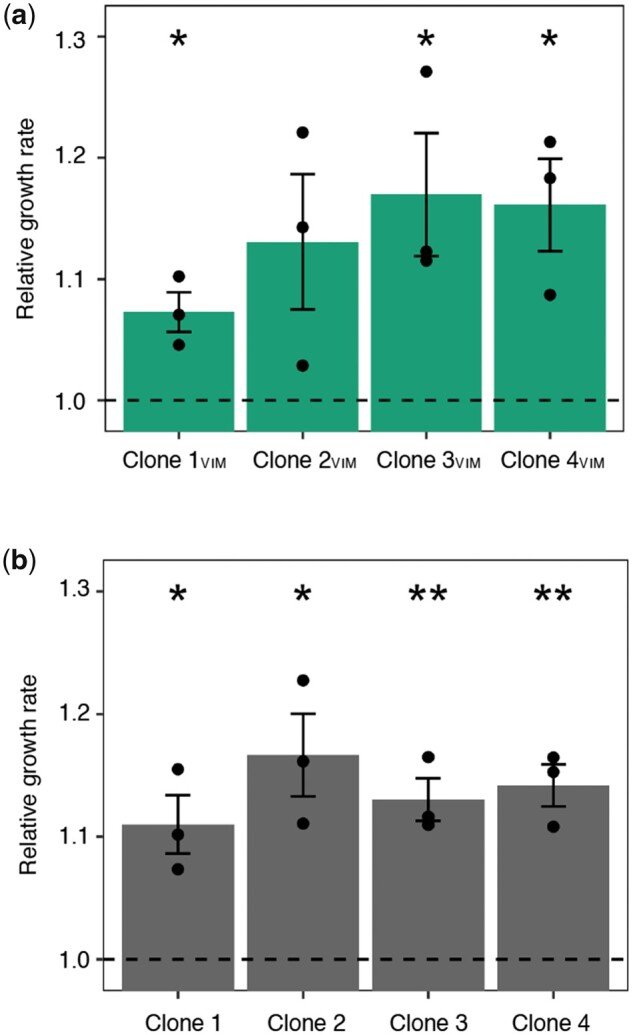
Fitness-improved adapted backgrounds. Exponential growth rates of (*a*) coevolved pG06-VIM-1-carrying strains relative to strain ExPEC+VIM and (each comparison *n* = 3) (*b*) coevolved pG06-VIM-1 segregants relative to ancestral strain ExPEC (each comparison *n* = 3). Significant fitness changes are indicated by asterisks (*P *=* ** < 0.05, ** < 0.01, *** < 0.001; one-sample *t*-test, one-sided). Error bars indicate ±SEM.

To verify that the mutations identified in the two regulatory systems increase fitness in the given *in vitro* environment, we assessed the fitness of pG06-VIM-1-containing and -free, evolved and ancestral strains by measuring exponential growth rates. Plasmid pG06-VIM-1 in Clones 1–4_VIM_ displayed no mutations after experimental evolution and this approach allowed us to directly measure the effects of the chromosomal mutations on general fitness. Growth rates of evolved strains were increased by 7–17% across all comparisons independent of presence or absence of the plasmid (one-sample *t*-test, one-sided: Clone 1: *w *=* *1.11 ± 0.02; *P *=* *0.022; Clone 2: *w *=* *1.17 ± 0.03; *P *=* *0.019; Clone 3: *w *=* *1.13 ± 0.02; *P *=* *0.009; Clone 4: *w *=* *1.14 ± 0.02; *P *=* *0.007; Clone 1_VIM_: *w *=* *1.07 ± 0.02; *P *=* *0.023; Clone 2_VIM_: *w *=* *1.13 ± 0.06; *P *=* *0.072; Clone 3_VIM_: *w *=* *1.17 ± 0.05; *P *=* *0.040; Clone 4_VIM_: *w *=* *1.16 ± 0.04; *P *=* *0.026; fig. 5*a* and *b*). Despite lower resolution than competition experiments, these data show that the identified mutations increase fitness under the given growth conditions and independent of plasmid carriage. They provide further support for the plasmid cost mitigating role of the observed chromosomal mutations.

### Transcriptional Alterations Contribute to Reduced Plasmid Costs

**Fig. 6.**
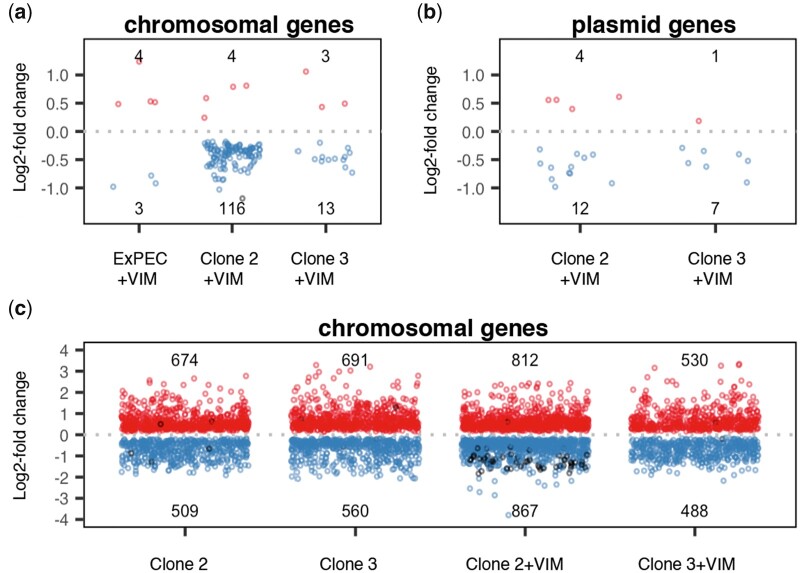
Differential expression analysis. Number of up- and downregulated genes (log_2_-fold change) on (*a*) chromosomes of ancestral strain ExPEC+VIM, evolved Clone 2+VIM and Clone 3+VIM upon acquisition of native pG06-VIM-1 (compared with respective plasmid-free strain) (*b*) evolved pG06-VIM-1 of Clone 2+VIM and 3+VIM due to adaptive chromosomal mutations (compared with ancestral ExPEC+VIM) (*c*) chromosomes of evolved pG06-VIM-1-free/-carrying Clone 2 and Clone 3 due to adaptive chromosomal mutations (compared with ExPEC and ExPEC+VIM, respectively). Circles: up (red) and downregulated (blue) protein-encoding genes; differentially regulated RNA-encoding genes (black).

CRP and ArcA represent two of seven global transcription factors in *E. coli* and directly or indirectly control the expression of several hundred genes (Liu and De Wulf 2004; Shimada et al. 2011). Changes in gene expression may lead to a reduced burden of plasmid carriage as demonstrated previously (Harrison et al. 2015; San Millan et al. 2015; Kawano et al. 2020). We sought to elucidate both the origin of the initial pG06-VIM-1 cost and its amelioration due to mutations in CCR- and ArcAB-associated genes and performed RNA-Seq. Six replicate samples of plasmid-free strains ExPEC, Clone 2 and Clone 3, and plasmid-carrying strains ExPEC+VIM, Clone 2+VIM, Clone 3+VIM were sequenced resulting in on average 25 million paired-end reads per sample (supplementary table 10, Supplementary Material online). Comparing the two ancestral strains ExPEC and ExPEC+VIM revealed differential expression of seven chromosomal genes immediately upon plasmid acquisition, of which only the one encoding a putative selenium delivery protein displayed a fold-change (2.36) beyond a 2-fold threshold (fig. 6*a* and supplementary table 11, Supplementary Material online). Similarly, plasmid pCAR1 also significantly changes the expression of only a limited set of chromosomal genes in *Pseudomonas putida*, but with a stronger effect (>40-fold) on only one gene designated *parI* (Miyakoshi et al. 2007). The lack of substantial evidence for altered chromosomal gene expression in our work suggests that the costly plasmid acquisition may not severely disrupt transcriptional regulation (Buckner et al. 2018) or specific cellular pathways, for example, SOS response (San Millan et al. 2015). Instead, the cost can derive from the usage of building blocks or molecular machinery for plasmid replication (e.g., nucleotides), but most likely those required for expression and posttranslational events, such as amino acids, ribosomes, chaperones, and acetyl/succinyl modification (San Millan et al. 2018; Vasileva et al. 2018 and reviewed in Baltrus 2013).

Given that the CCR and ArcAB systems are involved in global gene regulation it is not surprising that mutations in evolved Clone 2 and 3 lead to considerable changes in chromosomal gene expression when compared with the ancestral ExPEC strain. Indeed, hundreds of genes are differentially up and downregulated independently of pG06-VIM-1 presence (fig. 6*c* and supplementary table 11, Supplementary Material online). Despite some differences among the four evolved clones, enrichment (supplementary table 12, Supplementary Material online) and overrepresentation (supplementary table 13, Supplementary Material online) analyses of protein-encoding genes show common trends; cell motility via cilia/flagella (and other processes that require cell component organization, such as the expression of adhesion factors) tends to be downregulated (supplementary fig. 5, Supplementary Material online), whereas there is upregulation of diverse metabolic processes that target macromolecule biosynthesis (e.g., amino acids) and ribosome assembly which are, directly or indirectly, connected to translation and gene expression (supplementary fig. 4, Supplementary Material online).

After evolution, only 16 chromosomal genes of Clone 3 were affected by pG06-VIM-1 acquisition, of which one gene encoding a phage tail protein, exhibited overexpression >2-fold (fig. 6*a* and supplementary table 11, Supplementary Material online). In Clone 2, we found upregulation of four chromosomal genes, whereas 116 were downregulated (fig. 6*a* and supplementary table 11, Supplementary Material online). Analyzing the 115 downregulated protein-encoding genes revealed an overrepresentation of biological processes involved in tRNA metabolism and nucleotide biosynthesis (supplementary table 13 and fig. 6, Supplementary Material online), whereas the remaining gene encodes a tRNA. Furthermore, the comparison Clone 2+VIM versus ExPEC+VIM revealed that 52 (or 65%) of the strain’s tRNA-encoding genes are downregulated (fig. 6*c* and supplementary table 11, Supplementary Material online) which is indicative of altered translation processes. Therefore, at least in Clone 2 the low cost of pG06-VIM-1 can be attributable to interference in translation, which is in agreement with other reports showing that low plasmid costs are associated with gene expression (McNally et al. 2016; Buckner et al. 2018).

Interestingly, overall expression of pG06-VIM-1 genes decreased in evolved hosts, such that in Clone 2+VIM 12 plasmid genes are downregulated and four upregulated, whereas in Clone 3+VIM, seven plasmid genes are downregulated but only one is upregulated (fig. 6*b* and supplementary table 11, Supplementary Material online). Although transcriptional changes in these genes never exceed a 2-fold threshold, the net fold-change is negative (−13.08 for Clone 2+VIM and −9.01 for Clone 3+VIM; supplementary table 17, Supplementary Material online). Plasmid RNA represents 2.33 ± 0.11% of the total transcripts for ExPEC+VIM, but a significantly lower proportion for evolved strains (one-way ANOVA assuming equal variances, df* *=* *2, *P *=* *0.001, followed by Dunnett’s test: *P *<* *0.001 and *P *=* *0.03), respectively 2.08 ± 0.08% for Clone 2+VIM and 2.18 ± 0.11% for Clone 3+VIM (supplementary fig. 7, Supplementary Material online). Taken together, these data suggest that net downregulation of plasmid genes after evolution offer a plausible explanation for the reduced fitness costs, whereas in other works, such reduction is explained by downregulation of highly specific plasmid genes (San Millan et al. 2015). Although the differences in the proportions of plasmid transcripts among hosts are small, they can lead to significant fitness effects if the synthesized proteins require chaperones (Ma et al. 2018) and posttranslational modification (Vasileva et al. 2018). Thus, reshaping gene expression at a global level through the identified mutations in the CCR and ArcAB systems affects plasmid transcription levels. This represents a novel solution to the plasmid paradox where adaptation to a new niche (growth medium in this case) pleiotropically mediates plasmid cost reductions.

## Discussion

In this report, we asked if and how plasmid–host coevolution would mitigate the fitness costs of two clinically highly relevant MDR plasmids newly acquired by a plasmid-free ExPEC isolate. Our data show that the moderate initial costs of both carbapenemase-encoding plasmids were significantly alleviated during laboratory evolution. Curiously, the main causes for amelioration were not plasmid-specific compensatory mutations as reported in several recent studies (San Millan et al. 2014; Harrison et al. 2015; Loftie-Eaton et al. 2016, 2017), although deletions of costly plasmid regions (Turner et al. 2014; Porse et al. 2016) and undetected plasmid mutations could also have played a role. Instead, after approximately 300 generations, we identified strong parallel evolution in chromosomal genes only, independent of plasmid carriage. The mutational target genes represented two global regulatory systems involved in *E. coli* carbon catabolite repression (CCR) and aerobic respiration (ArcAB). Moreover, the mutations in these transcriptional regulators improved the maintenance of two unrelated plasmids strongly suggesting that the ExPEC host became generally more permissive toward plasmid acquisition, and in the future, it would be interesting to test the effect on additional plasmid types. The pleiotropic effects on plasmid cost amelioration appear to be mainly due to mutations affecting the CCR regulatory system, as demonstrated by fitness results using *cpdA* and *crp* deletion mutants. Mechanistically, RNA-seq analyses revealed a net transcriptional relief on plasmid genes as a collateral cost-mitigating effect of environmental adaptation by global regulatory changes.

Other studies have also reported that mutations in regulatory systems improved plasmid–host relationships. In a seminal study, mutations in the *gacA/gacS* two-component regulatory system reduced the cost of the mega plasmid pQBR103 by decreasing plasmid transcriptional demand in *Pseudomonas fluorescens* (Harrison et al. 2015). These mutations were specifically ameliorating the cost of the plasmid since they did not appear in the plasmid-free evolved lineages (true compensatory mutations) (Harrison et al. 2015). This is categorically different from our findings since we observed that adaptation in the CCR and ArcAB regulatory systems was not specific to plasmid-carrying populations. Two other reports frequently identified mutations in regulatory systems across multiple plasmid-carrying evolving populations that improved plasmid maintenance (Loftie-Eaton et al. 2016; Stalder et al. 2017). However, the absence of evolved plasmid-free lineages in these studies precludes direct comparisons with the results presented here.

On a broader perspective, our results warrant further research on plasmid-evolutionary dynamics in different *E. coli* lineages and sublineages to better understand why some of them appear to be more prone to acquire and maintain MDR plasmids (Mathers et al. 2015; McNally et al. 2016). Available data support that plasmids of clinical origins rarely reduce fitness of clinical host strains (Sandegren et al. 2012; Schaufler et al. 2016; Di Luca et al. 2017; Buckner et al. 2018; Gama et al. 2020; Ma et al. 2020) to the extent often seen in the pioneering studies of plasmid–host compensatory evolution (Lenski et al. 1994; Sota et al. 2010; San Millan et al. 2014; Harrison et al. 2015). It is also clear that this alone cannot explain how successful clone–plasmid associations emerge. From population genomic analyses McNally and coworkers demonstrated an association between mutations in regulatory regions of the high-risk *E. coli* ST131 subclade C and the accessory genome, including MDR plasmids. In their interpretation, this finding represented evidence of compensatory evolution toward MDR plasmid acquisition. However, taken together with a recent report showing that ExPEC ST131 has adapted to separate ecological niches at the subclade level, our results provide an alternative explanation (McNally et al. 2019).

Based on data presented here, it can be hypothesized that regulatory changes could also in part represent niche adaptations that coincidentally facilitate MDR plasmid acquisition and maintenance. Moreover, chromosomal antibiotic-resistance mutations, which can be viewed as a form of niche adaptation, display epistatic interactions affecting plasmid fitness cost (Silva et al. 2011) as well as stability (Sota and Top 2008). Therefore, different types of mutations causing environmental adaptation can collaterally increase the permissiveness to plasmids. However, we acknowledge that the pleiotropic effects on plasmid costs reported here may be specific to a single environment, as others have reported that both fitness costs (Knoppel et al. 2018; San Millan et al. 2018; Hubbard et al. 2019) and compensatory evolution (Hall et al. 2020) are highly media-dependent. Consequently, the specific mutations reported here may be media-dependent, but the processes targeted (i.e., global gene regulation) are widely reported across different media, strains, and plasmids supporting the generality of our findings.

Our study is not without limitations. We specifically dissected the causes of plasmid fitness cost amelioration for the nonconjugative pG06-VIM-1, but not in detail for pK71-77-1-NDM. Maintenance of the latter improved due to mutations in CCR/ArcAB regulons, but the contribution of each system remains unclear. Due to our experimental approaches, we could not precisely identify whether the chromosomal mutations affected fitness directly, or indirectly due to observations that conjugative transfer increased in the evolved clones. To that end, ArcA has been shown to impact conjugative transfer of other plasmid types (Strohmaier et al. 1998; Serna et al. 2010). However, we cannot exclude the possibility that the small increase in pK71-77-1-NDM conjugation is an artifact resulting from different growth rates, since adapted donors display faster growth than unevolved donor and recipient strains. The effects of CCR/ArcAB mutations on the different parameters for pK71-77-1-NDM maintenance (fitness costs, stability, and conjugation) need further exploration. In addition, the role of large plasmid deletions represents a subject for future research.

In this report, we propose “piggybacking” on niche adaptation as a novel, not mutually exclusive, solution for the “plasmid paradox.” Our approaches also underscore the importance of using clinically relevant strains and plasmids to investigate the evolutionary dynamics of plasmid-mediated antibiotic resistance. This knowledge can be used jointly with data from molecular epidemiology to better predict future emergence of successful combinations of clones, sublineages, and antibiotic resistance determinants.

## Materials and Methods

### Bacterial Hosts, Plasmids, and Culture Conditions

Strains, plasmids, and primers used in this study are listed in supplementary tables 1 and 2, Supplementary Material online. The ancestral plasmid-free strain ExPEC was chosen as it represents a clinically relevant *E. coli* isolate (originating from urinary tract infection) while being plasmid-naïve. It belongs to sequence type 537, as tested by multilocus sequence typing, and phenotypically susceptible to 24 antibiotics tested by disc diffusion (Bengtsson et al. 2012; Kahlmeter and Poulsen 2012) (supplementary table 1, Supplementary Material online). The lack of detected replicons and antibiotic resistance genes make this strain an ideal clinical model to study the behavior MDR plasmids. Plasmids pG06-VIM-1 and pK71-77-1-NDM originated from a *K. pneumoniae* wound infection isolate (Samuelsen et al. 2011) and an uropathogenic *E. coli* (Samuelsen et al. 2011) and were introduced into ExPEC by electroporation or conjugation, respectively. Strains were grown at 37°C under aeration in Miller Difco Luria–Bertani liquid broth (LB; Becton, Dickinson and Co.) or on LB agar (LBA) containing additional Select agar (15 g l^−1^, Sigma–Aldrich). For selection of plasmid-carrying strains, media were supplemented with ampicillin (100 mg l^−1^; Sigma–Aldrich). See supplementary section I, Supplementary Material online, for more details on strains constructed in this study.

### Experimental Evolution

Single colonies of strains ExPEC, ExPEC+VIM, and ExPEC+NDM were used to initiate four independent lineages each. The 12 lineages were evolved in 1 ml of antibiotic-free LB medium using 2 ml-deep-96-well plates in checkered pattern (VWR International) and incubated at 37°C with 700 rpm constant shaking (Microplate Shaker TiMix 5, Edmund Bühler). In total, 48 transfers with estimated 6.6 generations between two transfers (∼300 generations) were performed involving a 1:100 dilution of stationary-phase cultures into fresh LB every 12 h (∼10^7^ cells transferred). Endpoint populations (Pop 1–4_VIM_, Pop 5–8_NDM_, and Pop 9–12) and one representative clone per plasmid-carrying evolved population (Clones 1–4_VIM_ and Clones 5–8_NDM_) were stored at −80°C (supplementary section I and supplementary table 1, Supplementary Material online).

### Whole-Genome Sequencing

See supplementary section II, Supplementary Material online, for details on long-read sequencing and assembly of a closed reference genome of strain ExPEC (GenBank accession number CP053079). For Illumina whole-genome sequencing, genomic DNA of ancestral strains ExPEC, ExPEC+VIM, ExPEC+NDM, eight evolved clones (Clones 1–4_VIM_, Clones 5–8_NDM_) and 12 evolved mixed populations (Pop 1–4_VIM_, Pop 5–8_NDM_, Pop 9–12) (fig. 1) was isolated using the GenElute Bacterial Genomic DNA Kit (Sigma–Aldrich). DNA-purity and -quantity was assessed using a NanoDrop ND-1000 spectrophotometer (Thermo Scientific). Short-read sequencing library preparation and sequencing were performed following manufacturers’ instructions at the Genomic Support Centre Tromsø, UiT The Arctic University of Norway. The Nextera XT DNA Library preparation kit (Illumina) was used with an input of 1 ng genomic DNA and dual indexes. Samples were sequenced on a NextSeq 550 instrument (Illumina) with 300 cycles (2 × 150 bp paired-end reads), and a NextSeq 500/550 mid-output flow cell was used for clonal samples. One entire high-output flow cell was explicitly used for the population samples aiming at deep coverage. We ran Trim Galore v0.5.0 with default settings to remove adapter sequences (CTGTCTCTTATA) and low-quality bases, and SPAdes v3.13.0 with read error correction (Bankevich et al. 2012; Krueger 2012). Trimmed and error-corrected short reads were controlled for adapters and quality score using FastQC v0.11.4 (Andrews 2010). The raw sequence reads (long and short) of 24 libraries are available from the NCBI Sequence Read Archive (SRA, BioProject accession number PRJNA630076).

### Short-Read Sequence Analysis

We used the breseq computational pipeline v0.33.0 and v0.35.0 for prediction of mutations from clonal and population short-read sequencing data (Deatherage and Barrick 2014). Preprocessed reads (see above) of all evolved populations were mapped against the reference genome of strain ExPEC (GenBank accession number CP053079), and against plasmid sequences of pG06-VIM-1 (Pop 1–4_VIM_; GenBank accession number KU665641; Di Luca et al. 2017) or pK71-77-1-NDM (Pop 5–8 _NDM_; GenBank accession number CP040884; Gama et al. 2020) when appropriate. Breseq was run with default settings except for specifications when analyzing clonal sequencing data (“consensus-mode”; “frequency-cutoff 0.9”; “minimum-variant-coverage 10”; “consensus-minimum-total-coverage 10”) or population sequencing data (“polymorphism-mode”; “frequency-cutoff 0.01”; “minimum-variant-coverage 10”; “minimum-total-coverage 100”; “base quality score 20”). We focused on the identification of de novo single nucleotide substitutions, deletions, insertions, and small indels by manually evaluating the predicted mutations from the breseq outputs (supplementary section IIIb, Supplementary Material online). The use of short-read sequencing data bears an inherent limitation regarding the interpretation of chromosomal inversions, rearrangements, and mutations in repeat regions due to misaligned reads, and these mutations were thus omitted from further analysis. Repeats were confirmed using “Tandem repeats finder” v4.09 (Benson 1999) or by manually searching the reference genome for multiple alignment options. For population sequencing analysis, we report genetic changes as low as 1% mutation frequency considering that the mutated locus reached ≥ 10% mutation frequency at least in one of the evolved populations. Artemis v16.0.0 (http://sanger-pathogens.github.io/Artemis/), Gene Construction Kit v4.0.3 (Textco Biosoftware Inc.), and the Integrative Genomics Viewer v2.6.0 (http://software.broadinstitute.org/software/igv/) were used to support manual inspection of sequencing data.

### Competitive Fitness and Plasmid Stability

The relative competitive fitness (*w*) of plasmid-carrying clones was determined in pairwise serial competition experiments (∼40 generations) with the isogenic plasmid-free strain, as described before (Starikova et al. 2013), with minor modifications. Briefly, preadapted cultures of each competitor were adjusted to the same OD_600_, mixed in a 1:1 ratio, and used to initiate 1 ml batch cultures at a density of approximately 10^7^ CFU (=*T*_0_), in antibiotic-free LB and 2 ml-deep-96-well plates in checkered pattern (VWR International). Plates were incubated at 37 °C with 700 rpm shaking (Microplate Shaker TiMix 5, Edmund Bühler), and the cultures were diluted 1:100 into fresh LB every 12 h (=*T*_12–72_). To determine the CFU of each competitor, cultures were diluted in 0.9% saline (m/v) and plated selectively on LBA-ampicillin (CFU_plasmid-carrying_) and nonselectively on LBA (CFU_total_) at *T*_0_ and every following timepoint. The selection coefficient was calculated as *s = *0.5×*b*/ln(1/*d*) with *b* (=slope) obtained from regressing the natural logarithm of the ratio (CFU_plasmid-carrying_/CFU_plasmid-free_) over timepoints, and *d* as the dilution factor at each transfer (here 1:100) (Levin et al. 2000). It was multiplied by 0.5 to account for two transfers per day (to obtain *s* per day). Relative fitness was calculated as *w = *1* *+* s*, where the fitness of the plasmid-free strain equals 1 (supplementary table 6, Supplementary Material online). To determine spontaneous plasmid loss during competition experiments, we proceeded similarly with preadapted cultures of plasmid-carrying strains as described above. Briefly, the density at *T*_0_ was approximately 5 × 10^6^ CFU ml^−1^ and cultures were transferred, diluted, and plated selectively and nonselectively, as described above. The slope obtained by regressing the frequency of the plasmid-carrying population (CFU_plasmid-carrying_/CFU_total_) over timepoints was calculated (supplementary table 7, Supplementary Material online). For determination of relative competitive fitness and spontaneous plasmid loss, results were obtained from at least three biological replicates, initiated on separate days, with three technical replicates each.

### Exponential Growth Rates

As a proxy for fitness changes due to acquired mutations in evolved clones with and without the plasmid, the exponential growth rates of separately growing strains were determined. Briefly, overnight cultures in 1 ml LB were started from a single colony grown on LBA, diluted 1:100 in LB, and 250 μl were aliquoted into a 96-well-microtiter plate (Thermo Scientific). Absorbance at OD_600_ nm was measured in a BioTek EPOCH2 microtiter spectrophotometer (BioTek Instruments), every 10 min, and with linear shaking. Growth rates (*r*) were determined using GrowthRates v3.0 (Hall et al. 2014). Fitness of the evolved strain was calculated as relative growth rate* *=* r*_evolved strain_/*r*_ancestral strain_. Results were obtained from three biological replicates including five technical replicates all displaying a correlation coefficient *R* ≥ 0.97.

### Intracellular cAMP Concentration

Intracellular cAMP was quantified using the cAMP Select ELISA Kit (Cayman Chemical) following manufacturers’ instructions. For this purpose, overnight cultures were started from single colonies into 2 ml LB, diluted 1:100 into fresh LB, and incubated until midexponential growth phase (between 5.3 and 6.7 × 10^8^ CFU ml^−1^). About 5 ml of each culture was spun down at 4 °C, 4,000 rpm for 10 min (Eppendorf Centrifuge 5810), supernatant was removed, and the pellet was subsequently washed three times in ice-cold 0.9% saline (m/v). Cells were resuspended in 250 μl of 0.1 M HCl to stop endogenous phosphodiesterase activity and the suspensions were boiled for 5 min. After centrifugation at 4 °C, 4,000 rpm for 10 min (3000 x *g*) an aliquot of the supernatant was diluted 1:2 (ExPEC, ExPEC+VIM) or 1:8 (Clone 2_VIM_, Clone 2) in ELISA buffer followed by a subsequent 1:2 dilution step for all samples. The cAMP standard was reconstituted in 0.1 M HCl but thereafter diluted into ELISA buffer. Samples were applied in two dilutions, each in three biological and three technical replicates on the same ELISA plate. The standard was applied once and in two technical replicates on the same plate. The plate was incubated in the dark for 18 h at 4 °C and thereafter developed under slow orbital shaking and dark conditions. Absorbance was measured at 410 nm periodically in a BioTek EPOCH2 microtiter spectrophotometer (BioTek Instruments). Final reads were taken at *B*_0–average_* *=* *0.7 and data were analyzed using the spreadsheet available at https://www.caymanchem.com/analysisTools/elisa (*R*^2^ standard curve* *=* *0.96).

### Total RNA Isolation

For transcriptome analysis, overnight cultures were initiated from single colonies into 2 ml LB, diluted 1:100 into fresh LB, and incubated until midexponential growth phase (OD_600_ 0.5–0.6; average 2.2 ± 0.9 × 10^8^ CFU ml^−1^). Total RNA was isolated in six biological replicates per strain from 0.5 ml of culture using the RNeasy Protect Bacteria Mini kit (Qiagen) on six consecutive days. RNA-quality and -quantity were assessed with Nanodrop ND-1000 spectrophotometer (Thermo Scientific). Contaminating genomic DNA (gDNA) was digested following rigorous DNAse I treatment of the Ambion DNA-free DNase kit (Thermo Scientific). Briefly, 50 μl assays of maximum 10 μg RNA were treated in two consecutive incubation steps at 37 °C for 30 min and addition of 5 μl DNase I enzyme before each step. RNA-quality and -quantity were again assessed as described above and the absence of gDNA was tested by PCR amplification (40 cycles) of the *adk* housekeeping gene (supplementary table 2, Supplementary Material online). The RNA integrity numbers (RIN) were obtained via the Agilent RNA 6000 Nano kit and the Agilent 2100 Bioanalyzer system (Agilent Technologies 2100), and all samples reached RIN >9 (supplementary table 10, Supplementary Material online). Depletion of ribosomal RNA from 1 μg total RNA per sample with the QIAseq FastSelect RNA Removal kit and library preparation using the Truseq Stranded mRNA library kit were performed at Qiagen (Genomic Service Hilden, Germany). The Norwegian Sequencing Centre (NSC) (http://www.sequencing.uio.no) performed sequencing of the library on 1/2×SP Novaseq flow cell with 300 cycles (2 × 150 bp paired-end reads). The raw sequence reads of 36 libraries are available from NCBI SRA (BioProject accession number PRJNA630076).

### RNA-Seq Analysis

NSC performed initial filtering of raw reads including adapter trimming and removal of low-quality reads using BBMap v34.56 (therein BBDuk) (Bushnell 2014). NSC mapped clean and adapter removed reads against the merged version of the ExPEC chromosome and the pG06-VIM-1 sequence using Hisat2 v2.1.0 (Kim et al. 2019) and generated count tables using FeatureCounts v1.4.6-p1 (Liao et al. 2014), resulting in an average sample alignment of 65% (supplementary table 10, Supplementary Material online). Count tables were used as input for the differential expression analysis (data normalization and statistical tests) performed in R version 4.0.2 (R Core Team 2018) using the default script for SARTools version 1.7.3 (Varet et al. 2016) with default settings and strain ExPEC as reference.

PANTHER Generic Mappings of chromosomal genes were generated using the PANTHER HMM Scoring tool with the PANTHER HMM library Version 15.0 (Ashburner et al. 2000; Mi, Muruganujan, Ebert, et al. 2019; Mi, Muruganujan, Huang, et al. 2019; Gene Ontology Consortium 2019; supplementary table 16, Supplementary Material online) and functional classification of the PANTHER accessions was retrieved from the website (supplementary table 15, Supplementary Material online). Tabular lists containing the gene ID, PANTHER accession, and fold change for all differentially expressed chromosomal genes were uploaded as PANTHER Generic Mappings to http://pantherdb.org/ and ran for enrichment of PANTHER GO-Slim Biological Processes with FDR (false discovery rate) correction. Enrichment analysis (supplementary table 12, Supplementary Material online) was performed for each of the comparisons in supplementary table 11, Supplementary Material online, except ExPEC+VIM versus ExPEC. For the overrepresentation analyses, subsets of the same lists (up and downregulated genes only) were uploaded separately to http://pantherdb.org/. Overrepresentation analyses (supplementary table 13, Supplementary Material online) were performed against the PANTHER Generic Mapping of all chromosomal protein-encoding genes (supplementary table 14, Supplementary Material online), with Fisher exact test and FDR correction. All GO terms displaying significant enrichment or overrepresentation were compiled in three subsets (up and downregulated processes independent of plasmid presence, and downregulated processes due to plasmid presence) and used to generate supplementary figures 4–6, Supplementary Material online, respectively, in Visualize (Day-Richter et al. 2007) available at http://amigo.geneontology.org/visualize?mode* *=* *client_amigo.

### Statistical Analyses

Statistical analyses were performed in R version 4.0.2 (R Core Team 2018). Samples were verified for normality with Shapiro–Wilk test and/or graphical visualization. Homogeneity of variances was tested with Levene’s test (from package car; Fox and Weisberg 2019) and/or graphical visualization. One-sample or two-sample comparisons were performed with Student’s *t*-tests. Packages sandwich (Zeileis 2004), car (Fox and Weisberg 2019), and multcomp (Hothorn et al. 2008) were required for ANOVA and multiple comparisons, respectively. Graphs in figures 1, 3, 4, 5, and 6 were produced with packages ggplot2 (Kassambara 2020), patchwork (Pedersen 2020), ggthemes (Arnold 2019), and RColorBrewer (Neuwirth 2014). Significance levels are indicated as: *P* value * < 0.05; ** < 0.01; *** < 0.001. Packages openxlsx (Schauberger and Walker 2019) and writexl (Ooms 2020) were used to read/write xlsx files. Packages data.table (Dowle and Srinivasan 2019) and jsonlite (Ooms 2014) were required to generate supplementary tables 11–16, Supplementary Material online.

## Supplementary Material

Supplementary data are available at *Molecular Biology and Evolution* online.

## Supplementary Material

msab091_Supplementary_Data
