# Supplementary material for: Piggybacking on Niche Adaptation Improves the Maintenance of Multidrug‐Resistance Plasmids
Source: Mol Biol Evol. 2021 Mar 24;38(8):3188–201. doi: 10.1093/molbev/msab091 (PMC8321521; doi:10.1093/molbev/msab091)
Supplement: msab091_Supplementary_Data [file msab091_supplementary_data.zip › MBE-20-1050_Kloos_Gama_Supplemental File 1_v2.pdf]

Supplemental file 1

Piggybacking on niche-adaptation improves the maintenance of multidrug resistance plasmids

Julia Kloos[1#] (ORCID: 0000-0002-8773-3463), João A. Gama[1#] (ORCID: 0000-0003-0967-6616), Joachim Hegstad[1,2] (ORCID: 0000-0001-9173-7195), Ørjan Samuelsen[1,3] (ORCID: 0000-0002-5525-2614), Pål J. Johnsen[1*] (ORCID: 0000-0002-6455-6433)

[1]Department of Pharmacy, Faculty of Health Sciences, UiT The Arctic University of Norway, Tromsø, Norway; [2]Department of Microbiology and Infection Control, University Hospital of North Norway, Tromsø, Norway; [3]Norwegian National Advisory Unit on Detection of Antimicrobial Resistance, Department of Microbiology and Infection Control, University Hospital of North Norway, Tromsø, Norway.

[#]These authors contributed equally to this work.

[*]Corresponding author:

UiT The Arctic University of Norway

Department of Pharmacy

Faculty of Health Sciences

9037 Tromsø

Norway

Phone: +47 47688974

e-mail: paal.johnsen@uit.no

Supplementary Information - Extended Methods and Results

I. **Strain construction:**

supplementary methods

II. **Long-read whole-genome sequencing and *de novo* reference assembly:**

supplementary methods and results

III. **Short-read whole-genome sequencing:**

supplementary methods

a. **Populations:** supplementary results

b. **Clones:** supplementary results

IV. **Competitive fitness and plasmid loss:**

supplementary results

V. **Measurement of pK71-77-1-NDM transfer frequency:**

supplementary methods and results

VI. **Antimicrobial susceptibility testing:**

supplementary methods and results

VII. **Protein function:**

supplementary methods and results

I. Strain construction.

See Supplementary Table 2 for all primers used during strain construction.

Supplementary methods:

Clones 1-4$_{VIM}$ and Clones 5-8$_{NDM}$: these strains are purified representatives of evolved, plasmid-carrying populations. Briefly, ampicillin-resistant colonies of increased size were screened for the presence of mutations in genes *arcA*, *arcB*, *cpdA* and *crp* by Sanger sequencing. For this purpose, colony PCR was performed using DreamTaq PCR master mix 2x (Thermo Scientific) with standard conditions. The PCR products were enzymatically purified using Exonuclease I (Thermo Scientific) and Shrimp Alkaline Phosphatase (rSAP; New England Biolabs). The purified amplicons were Sanger sequenced by BigDye 3.1 chemistry (Applied Biosystems) employing single primers of the respective primer-pair. The assays were analyzed in-house on an Applied Biosystems 3130*xl* Genetic Analyzer automated cycler.

Clones 1-4: these strains are spontaneous pG06-VIM-1-free segregants identified as ampicillin-susceptible isolates by screening co-evolved Clones 1-4$_{VIM}$ on LBA and LBA-ampicillin (Luria-Bertani agar plates without and with ampicillin). The presence of clone-specific mutations was confirmed by Sanger sequencing in Clones 1-4 as described above. However, no plasmid-free segregants of co-evolved Clones 5-8$_{NDM}$ were obtained using the same method. In Clone 2 and Clone 3, which were further used in strain constructions, we verified the absence of chromosomal mutations other than the clone-specific ones by Illumina sequencing.

Clone 2+VIM, Clone 3+VIM, Clone 12+VIM, ExPEC+evVIM, BW25113+VIM, BWΔ*cpdA*+VIM, BWΔ*arcA*+VIM and BWΔ*crp*+VIM: these strains were either evolved without plasmid (Clone 12), plasmid-free evolved segregants (Clone 2 and Clone 3), deletion

mutants from the Keio collection [1] or the ancestral ExpEC strain. They received either the ancestral or evolved, non-conjugative pG06-VIM-1 by electroporation. For this purpose, ancestral pG06-VIM-1 was isolated from ExPEC+VIM and evolved pG06-VIM-1 from Clone 2_{VIM}, following the Qiagen protocol for isolation of very low-copy number plasmids using 500 mL culture volume and Qiagen-tip 100 (Qiagen). Electroporation was performed according to [2] and transformants were selected on LBA-ampicillin. Presence of pG06-VIM-1 was verified by replicon PCR (*repB*) in all purified clones. Sanger sequencing (see above) confirmed clone-specific mutations (Clone 2+VIM and Clone 3+VIM). PCR amplification and gel electrophoresis confirmed the deletion constructs of *cpdA, crp* and *arcA* in the Keio strains.

ExPEC+NDM, Clone 2+NDM and Clone 3+NDM: these strains carried the ancestral pK71-77-1-NDM and were obtained through conjugation by filter mating. For this purpose, M9 Minimal Medium agar was prepared from 5×M9 salts (200 mL L^{-1}) [3], 20% arabinose (20 mL L^{-1}), 200 mM $CaCl_2$ (0.5 mL L^{-1}), 1M $MgSO_4$ (2 mL L^{-1}), Select agar (15 g L^{-1}) and ampicillin (100 mg L^{-1}) (all Sigma-Aldrich). Tetrazolium Arabinose agar (TAA) was prepared from peptone (10 g L^{-1}; Oxoid, now Thermo Scientific), Yeast extract (0.1 g L^{-}1; Fluka, now Sigma-Aldrich), NaCl (5 g L^{-1}; Sigma-Aldrich), Select agar (15 g L^{-1}; Sigma-Aldrich), 20% arabinose (50 mL L^{-1}; Sigma-Aldrich) and 5% 2,3,5-Triphenyltetrazolium chloride (1 mL L^{-1}; Sigma-Aldrich). Each mating experiment was started by mixing overnight cultures of donor and recipient strains in a 1:1 ratio (500 µl each), 1:10 up-concentration of the mix by centrifugation, and application on a sterile filter (Pall Laboratory, mixed cellulose esters membrane) placed on LBA. After 6-8 hours of incubation at 37°C, bacteria were harvested by vortexing the filter in 2 mL of sterile 0.9% saline (m/v). Appropriate dilutions of the suspension were selectively plated. Firstly, strain K71-77 [4] served as the donor for ancestral pK71-77-1-NDM into rifampicin-resistant recipient strain MG1655 Δ*ara* RifR [5]

generating strain MG1655 Δ*ara* RifR+NDM. Here, transconjugants carrying pK71-77-1-NDM were selected on LBA-rifampicin + ampicillin. A pure clone was verified for the inability to use arabinose as sole carbon source by a red colony phenotype on TAA. Presence of pK71-77-1-NDM (*bla*$_{NDM-1}$) and absence of pK71-77-2 (IncFIA-replicon) were confirmed by PCR. Secondly, MG1655 Δ*ara* RifR+NDM was employed as counter selectable pK71-77-1-NDM donor strain to generate transconjugants ExPEC+NDM, Clone 2+NDM and Clone 3+NDM, which were selected on M9 Minimal Medium agar containing arabinose and ampicillin. Pure clones were verified for their ability to use arabinose by a white colony phenotype on TAA, and by confirming the presence of pK71-77-1-NDM (*bla*$_{NDM-1}$) and clone-specific mutations by PCR and Sanger sequencing as described above.

II. Long-read whole-genome sequencing and *de novo* reference assembly.

Supplementary methods:

Long-read sequencing was performed on ancestral strain ExPEC (Supplementary Table 1) using Pacific Biosciences technology (Pacific Biosciences) at the Norwegian Sequencing Centre (http://www.sequencing.uio.no). For this purpose, genomic DNA was isolated from 4.5×10^9 cells (3.75 mL of a 4.0 McFarland suspension) of an overnight culture started from a single colony grown on LBA. Qiagen 100/G Genomic tips were used following the manufacturers' protocol for isolation of genomic DNA from Gram-negative bacteria (Qiagen). DNA-purity and -quantity were determined using NanoDrop ND-1000 spectrophotometer (Thermo Scientific). Additionally, DNA-quantity was assessed using Qubit High Sensitivity DNA assay (Thermo Scientific) and DNA-integrity was assessed on a 1% agarose gel. Long-read sequencing was performed using single-molecule real-time (SMRT) sequencing technology according to the Pacific Biosciences protocol with PacBio Barcoded Adapters for Multiplex Sequencing. The DNA was sheared to 20 kb fragments

using Megaruptor (Diagenode). The final library was size selected using BluePippin (Sage Science) with 10 kb cutoff. The library was sequenced on a Pacific Biosciences Sequel instrument using Sequel Polymerase v3.0, SMRT cells v3 LR and Sequencing chemistry v3.0. Reads were demultiplexed using Barcoding pipeline on SMRT Link (v6.0.0.47841, SMRT Link Analysis Services and GUI v6.0.0.47836) with 26 as minimum barcode score. Sequences were assembled using HGAP4 assembly pipeline on SMRT Link (v6.0.0.47841, SMRT Link Analysis Services and GUI v6.0.0.47836) with default settings and 5 MB as expected genome size.

We trimmed and downsized the long-read data to a total of 500 Mb using Filtlong v0.2.0 [6] whereby 10% of the worst-quality reads were discarded and the longest reads were maintained. A circularized reference genome of strain ExPEC was generated by *de novo* hybrid assembly of short- (see Supplementary information section IIIb) and long-read sequencing data using Unicycler v0.4.7 [7] with default settings. The circularized genome was corrected for single-nucleotide polymorphisms arising from alignment of the Illumina reads using Snippy v4.3.6 [8]. The final reference genome was annotated using the NCBI Prokaryotic Genome Annotation Pipeline [9] and is accessible at GenBank (accession CP053079).

III. Short-read whole-genome sequencing.

Supplementary methods:

Mutations from short-read sequencing data were predicted using the breseq computational pipeline v0.33.0 and v0.35.0 [10] as described in Methods. Further, breseq output files were manually screened; variants that were also predicted in the ancestral chromosome or plasmids when using the same alignment parameters (clonal or polymorphism mode) were excluded from downstream analysis. Moreover, variants in homopolymeric

regions or regions with ambiguous reference sequence due to poor sequencing coverage were excluded.

a) Populations.

Supplementary results:

We performed ‘deep’ short-read sequencing on all evolved populations (Pop 1-4$_{VIM}$, Pop 5-8$_{NDM}$, Pop 9-12). An average coverage of 1322×/1800× was reached for chromosomal or plasmid sequencing data, respectively (Supplementary Table 5). We did not observe signs of hypermutability from our population sequencing data and nonsense mutations were not reported for any of the evolved populations. In *cpdA* (3',5'-**c**yclic adenosine monophosphate (cAMP) **p**hospho**d**iesterase), genetic changes involved also small deletions (Δ1, Δ3, Δ4 and Δ13 bp) or short sequence insertions (+3, +4, +6 bp) (35%; Supplementary Table 4). Notably, mutations in *arcB* (**a**erobic **r**espiration **c**ontrol sensor protein; histidine kinase) were specific for one plasmid-free population (Pop 9) and three pK71-77-1-NDM-carrying populations (Pop 5$_{NDM}$, Pop 6$_{NDM}$ and Pop 7$_{NDM}$) (Supplementary Table 4). Details on variant position, type and frequency are listed in Supplementary Table 4.

The most abundant and usually also most frequent mutation present in CpdA was the deletion of three base pairs (TGC) in amino acid (aa) position 163 (*cpdA*.Δ3.bp488-490) in 7/12 evolved populations. This deletion resulted in the loss of one leucine in a string of four leucines (aa 160-163). CpdA activity depends on the binding of two iron cations as cofactors and we identified aa substitutions and deletions in positions that are reported to be involved in metal cation binding in 4/12 evolved populations at low frequency (1.1-12.5%; D22A, H24Y/R, H164del (= *cpdA*.Δ3.bp490-492), H203Y and H205Y) (UniProtKB-P0AEW4). Additionally, aa positions 24 and 205 of CpdA are described as binding sites for cAMP ligand

to the enzyme and were affected by mutations in 3/12 evolved populations (UniProtKB-P0AEW4).

The predominant mutation in CRP (**c**AMP **r**eceptor **p**rotein; DNA-binding transcriptional regulator), G142S, was present in 5/10 evolved lineages, in which it was also the most frequent. The C-terminal part of CRP represents the DNA-binding HTH-domain (aa positions 138-210) and seven of in total eight identified mutations were located in this region of the protein (UniProtKB-P0ACJ8). In CRP, a hinge region located in aa positions 136-139 connects the N- and C-terminal domains of the protein [11], and aa changes in positions 142, 145, 149 and 135 in close proximity to this region are described to influence the cAMP dependence of the protein, especially when replaced with polar amino acids, resulting in cAMP-independent CRP* mutants [12] [13] [14]. Harman *et al.* described a CRP* phenotype of *E. coli* for aa substitution L196R [15], a variant which was also identified here. We did not identify mutations in aa positions that are, to our knowledge, directly involved in cAMP binding to CRP.

In ArcA (**a**erobic **r**espiration **c**ontrol protein; DNA-binding transcriptional regulator), mutation L50Q was the major variant and present in 5/12 populations but only most frequent in population Pop 3_{VIM} (100%). In this protein, a receiver domain is described for aa positions 1-135 and a DNA binding domain for aa positions 135-238 (UniProtKB-P0A9Q1). The majority of mutations identified in ArcA was located in the receiver domain (22/25), and only three in the DNA-binding domain, similar to mutations identified in *arcA* of *E. coli* and *Citrobacter freundii* adapting to nutrient-rich conditions [16].

The mutations identified in ArcB were specific to single evolved populations, except aa substitution S280R, which occurred in two populations. The majority of aa substitutions was identified in described regions of ArcB such as the PAS-domain (S159F), the PAC-domain (R269C), the primary transmitter domain (A410T, F413C, I442T), the receiver

domain (L579W) and the secondary transmitter domain (L686F, F689V, L734R, C746W; UniProtKB-P0AEC3).

Additionally, single evolved populations obtained mutations in distinct chromosomal targets; a 15 bp-deletion in *polA* (DNA polymerase I; Pop 9) and a synonymous mutation in *yncE* (uncharacterized protein; Pop 4_{VIM}) (Supplementary Table 4). We found no indications that the mutation observed in Pop 2_{VIM} upstream of *cyaA* affected the genes' promoter region.

None of the co-evolved populations had acquired point mutations in plasmid sequences. However, the plasmid coverage plot of evolved population Pop 5_{NDM} displayed a considerable drop in read coverage depth for a ~8.8 kb region in pK71-77-1-NDM (Supplementary Figure 1) spanning from position ~77602 nt (*rmtC*; 16S rRNA-methyltransferase) to ~86464 nt (IS*1* family transposase) indicating a deletion. This plasmid segment contained an aminoglycoside resistance gene (*aac(3)-IId*), amongst other genes, but did not include the gene encoding the plasmids' carbapenemase (bla_{NDM-1}) (Supplementary Table 3).

b) Clones.

Supplementary results:

Short-read whole-genome sequencing of strains ExPEC, ExPEC+VIM, ExPEC+NDM, Clones 1-4_{VIM}, Clones 5-8_{NDM}, Clone 2 and Clone 3 resulted in an average coverage of 117×/142× for chromosomes and plasmids, respectively (Supplementary Table 5). Using short-read sequencing of strain ExPEC, MLST v2.0 [17], Plasmidfinder v2.0 [18] and Resfinder v4.0 [19] we confirmed that the reference strain belonged to sequence type 537 and was without plasmids or acquired antibiotic-resistance mutations. For all sequenced plasmid-carrying clones, pG06-VIM-1 and pK71-77-1-NDM copy-numbers were calculated by dividing plasmid coverage with chromosomal coverage obtained from short-read

sequencing analysis (Supplementary Table 5). Plasmid copy-numbers were unchanged after evolution (Clones 1-4$_{VIM}$ = 0.9-1.5, average = 1.1; Clones 5-8$_{NDM}$ = 1.2-1.5, average = 1.4; while 1.3 for ExPEC+VIM and ExPEC+NDM). Read alignment of Clone 5$_{NDM}$ against ancestral pK71-77-1-NDM confirmed an ∼8.8 kb deletion as described before for Pop 5$_{NDM}$ (Supplementary information section IIIa). Furthermore, an even larger plasmid deletion was observed from coverage plots for Clone 7$_{NDM}$ covering a 58.9 kb region from position ∼27596 nt (IS*1380*-like element; IS*Ec9* family transposase) to ∼86456 nt (IS*1* family transposase) (Supplementary Figure 1). The missing plasmid segment in Clone 7$_{NDM}$ included the region that was also deleted in Clone 5$_{NDM}$ in addition to genes of the adjacent deleted region (Supplementary Table 3). The deleted plasmid segments were flanked by mobile elements either on both sides (Clone 7$_{NDM}$; IS*1380* and IS*1*) or at one side (Clone 5$_{NDM}$; IS*1*) which suggests that recombination events involving these genetic elements lead to the deletion event.

IV. Competitive fitness and plasmid loss.

Supplementary results:

A summary of the results from linear regression analysis in serial competition experiments and from determination of plasmid loss is given in Supplementary Tables 6 and 7.

V. Measurement of pK71-77-1-NDM transfer frequency.

Supplementary methods:

12-hours-conjugation assays were performed to estimate the transfer of pK71-77-1-NDM during fitness measurements. Ancestral strain ExPEC+NDM and evolved Clone 2+NDM and Clone 3+NDM represented donors for mating cultures. Rifampicin-resistant

strain K56-43 RifR [4] was employed as recipient (Supplementary Table 1). Overnight cultures in LB were started for the respective strains and used to inoculate 1 mL LB with ~10^7 CFU mL^{-1} of donor and recipient cells (1:1) in a 2 mL-deep-96-well plate (VWR International) (= T_0). The plate was incubated at 37°C and 700 rpm shaking (Microplate Shaker TiMix 5, Edmund Bühler) for 12 hours (= T_{12}). Rifampicin (100 mg L^{-1}; Sigma-Aldrich) was added to LBA when appropriate. At T_0 and T_{12}, CFU_{donor} and $CFU_{recipient}$ were determined on LBA-ampicillin and LBA-rifampicin, respectively. At T_{12}, $CFU_{transconjugants}$ was additionally determined on LBA-rifampicin + ampicillin. The frequency (%) of transconjugants per recipient was calculated as:

$$\frac{CFU_{transconjugants}}{CFU_{recipient}} \times 100$$

Supplementary results:

Results were obtained from five biological replicates with three technical replicates each. The conjugation frequencies were 0.25% (ExPEC+NDM), 0.52% (Clone 2+NDM) and 0.75% (Clone 3+NDM), all significantly different from each other (one-way ANOVA assuming equal variances, df = 2, $P < 0.001$, followed by Tukey's test; Supplementary Figure 3).

VI. Antimicrobial susceptibility testing.

Coverage plots from clonal short-read sequencing analysis indicated that deletions had occurred in co-evolved pK71-77-1-NDM sequences of Clone 5_{NDM} and Clone 7_{NDM} (Supplementary information section IIIb and Supplementary Figure 1). For both clones, these deletions included aminoglycoside resistance genes (Supplementary Table 3). To phenotypically examine these predicted deletions, we compared changes in the susceptibility

of the ancestral strain ExPEC+NDM and the evolved Clones 5-8_{NDM} for two aminoglycoside antibiotics, tobramycin and gentamicin by disc diffusion.

Supplementary methods:

Antimicrobial susceptibility of ancestral strain ExPEC+NDM and evolved Clones 5-8_{NDM} was investigated by disc diffusion tests on Mueller Hinton II agar (MHA; Becton, Dickinson and Company). *E. coli* ATCC 25922 was included for quality control purposes. Briefly, 0.5 McFarland solutions in 0.9% saline (m/v) were prepared from freshly grown colonies on LBA, and the inocula were spread evenly onto MHA plates. One disc was applied per plate and strain for each antibiotic (10 µg per disc; Oxoid, now Thermo Scientific). Plates were incubated at 37°C and zone diameter was measured after 18 hours. Disc tests were interpreted (sensitive or resistant) according to EUCAST zone diameter breakpoints [20] (Supplementary Table 8). Measurements were repeated three times per drug and clone and we used the median of the results as final value for interpretation.

Supplementary results:

We found that strain ExPEC+NDM, Clone 6_{NDM} and Clone 8_{NDM} were fully resistant to these aminoglycosides, while Clone 5_{NDM} and Clone 7_{NDM} displayed increased susceptibility for both antibiotics. Both, Clone 5_{NDM} and Clone 7_{NDM} were now susceptible to gentamicin whereas only Clone 7_{NDM}, which was affected by the larger deletion in pK71-77-1-NDM, was also susceptible to tobramycin (Supplementary Table 8). The changed resistance levels of Clone 5_{NDM} and Clone 7_{NDM} confirmed that deletions of the described pK71-77-1-NDM-regions had occurred during experimental evolution, which were likely mediated by plasmid-encoded mobile elements as also observed by Porse *et al.* [21].

VII. Protein function.

Supplementary methods:

Three online tools (PROVEAN (http://provean.jcvi.org/index.php) [22], SIFT (https://sift.bii.a-star.edu.sg) [23] and SNAP2 (https://rostlab.org/services/snap2web/) [24] were used to predict the effect of non-synonymous mutations, identified from clonal and whole population sequencing, on CpdA, CRP, ArcA and ArcB protein function. To conclude on a potential effect of a mutation, two of three prediction tools had to meet the criteria for 'deleterious' (PROVEAN), 'effect'(SNAP2) or 'affect protein function'(SIFT).

Supplementary results:

A majority (76%) of the 59 examined mutations were predicted to have a deleterious effect on protein function, and each prediction was either supported by all three (67% or 30/45) or at least two prediction models (33% or 15/45, Supplementary Table 9). For single proteins, 93% of the mutations were predicted deleterious in CpdA, 83% in ArcB, 68% in ArcA, and 50% in CRP.

Supplementary Information - Tables and Figures

Supplementary Table 1. Plasmids and strains used in this study.

Supplementary Table 2. Primers used in this study.

Supplementary Table 3. Annotated genes present in deleted regions of evolved pK71-77-1-NDM.

Supplementary Table 4. Mutations identified in evolved populations.

Supplementary Table 5. Descriptive statistics from clonal and population short-read whole-genome sequencing data alignment to sequences of ancestral reference strain ExPEC and the respective plasmid.

Supplementary Table 6. Summary of the results from linear regression analysis (serial competition experiments).

Supplementary Table 7. Summary of the results from linear regression analysis (plasmid loss).

Supplementary Table 8. Results from susceptibility testing of ancestral and evolved pK71-77-1-NDM-carrying clones by disc diffusion test.

Supplementary Table 9. Effect of non-synonymous mutations on protein function in evolved populations.

Supplementary Tables 10-17 (Supplemental File 2; Excel sheet). Results RNA-Seq. RNA-Seq samples: quality and libraries statistics (Supp.Table 10); Differential Expression analysis (chromosome and plasmid) (Supp.Table 11); Enrichment analysis (chromosome) (Supp.Table 12); Over-representation analyses (chromosome) (Supp.Table 13); protein-encoding genes (chromosome) (Supp.Table 14); Functional Classification (chromosome) (Supp.Table 15); PANTHER Generic Mappings (Supp.Table 16); Net fold-change plasmid genes in Clone 2+VIM and Clone 3+VIM (Supp.Table 17).

Supplementary Figure 1. Read coverage plots of evolved pK71-77-1-NDM in evolved Pop 5_{NDM}, Clone 5_{NDM} and Clone 7_{NDM}.

Supplementary Figure 2. Number of chromosomal variants per evolved population and mutational target gene.

Supplementary Figure 3. Conjugation frequency of ancestral pK71-77-1-NDM from ancestral and evolved backgrounds into strain K56-43 RifR.

Supplementary Figures 4-6 (additional PDF files). Ontology graphs of enrichment and overrepresentation analysis RNA-Seq: upregulated processes in Clone 2 and 3 with and without pG06-VIM-1 due to adaptive mutations (Supp.Figure 4), downregulated processes in Clone 2 and 3 with and without pG06-VIM-1 due to adaptive mutations (Supp.Figure 5); downregulated processes due to plasmid presence in Clone 2+VIM (Supp.Figure 6).

Supplementary Figure 7. Proportion of plasmid RNA transcripts.

Supplementary Table 1. Plasmids and strains used in this study.

Plasmids[a]	Comment	Source
pG06-VIM-1	IncR; 53 kB; non-conjugative; *bla*$_{VIM-1}$, *aadA1, aadA2, aacA7, aphA, mphA, mphR, mrx, sul1, dfrA1, dfrA12, qacEΔ1*; originates from a *K. pneumoniae* wound infection isolate	[25], [26], GenBank KU665641
pK71-77-1-NDM	IncC; 145 kB; conjugative; *bla*$_{NDM-1}$, *bla*$_{CMY-6}$, *aac(6')-Ib, aac(3)-II, rmtC, sul1, ble*$_{MBL}$; originates from an uropathogenic *E. coli* isolate	[27], [4], GenBank CP040884

Strains/Populations[a]	Comment	Source	Identifier[b]
**Ancestral**			
ExPEC	*E. coli* K56-43; plasmid-free	[28], GenBank CP053079	MP04-43
ExPEC+VIM	ExPEC transformed with ancestral pG06-VIM-1; *E. coli* G1-15 in [26]	[26]	MP05-29
ExPEC+NDM	ExPEC transconjugant harbouring pK71-77-1-NDM	this study	MP16-39
**Evolved**			
Pop 1_{VIM}	co-evolved ExPEC+VIM lineage 1; mixed population	this study	MP16-01
Pop 2_{VIM}	co-evolved ExPEC+VIM lineage 2; mixed population	this study	MP16-02
Pop 3_{VIM}	co-evolved ExPEC+VIM lineage 3; mixed population	this study	MP16-03
Pop 4_{VIM}	co-evolved ExPEC+VIM lineage 4; mixed population	this study	MP16-04
Pop 5_{NDM}	co-evolved ExPEC+NDM lineage 1; mixed population	this study	MP16-05
Pop 6_{NDM}	co-evolved ExPEC+NDM lineage 2; mixed population	this study	MP16-06
Pop 7_{NDM}	co-evolved ExPEC+NDM lineage 3; mixed population	this study	MP16-07
Pop 8_{NDM}	co-evolved ExPEC+NDM lineage 4; mixed population	this study	MP16-08
Pop 9	evolved ExPEC lineage 1; mixed population	this study	MP16-09
Pop 10	evolved ExPEC lineage 2; mixed population	this study	MP16-10
Pop 11	evolved ExPEC lineage 3; mixed population	this study	MP16-11
Pop 12	evolved ExPEC lineage 4; mixed population	this study	MP16-12
Clone 1_{VIM}	selected clone of Pop 1_{VIM}; ArcA.M39V; CpdA.S222L	this study	MP16-13
Clone 2_{VIM}	selected clone of Pop 2_{VIM}; ArcA.L117Q; *cpdA.*Δ3.bp488-490	this study	MP16-14
Clone 3_{VIM}	selected clone of Pop 3_{VIM}; ArcA.L50Q; Crp.G142S	this study	MP16-15
Clone 4_{VIM}	selected clone of Pop 4_{VIM}; ArcA.F79Y; CpdA.P221L; YncE.T178T	this study	MP16-16
Clone 5_{NDM}	selected clone of Pop 1_{NDM}; ArcA.L93F; *cpdA.*Δ3.bp488-490	this study	MP16-17
Clone 6_{NDM}	selected clone of Pop 2_{NDM}; ArcA.M39I; *cpdA.*Δ3.bp488-490	this study	MP16-18
Clone 7_{NDM}	selected clone of Pop 3_{NDM}; ArcA.M53I; Crp.G142S	this study	MP16-19
Clone 8_{NDM}	selected clone of Pop 4_{NDM}; ArcA.M53I; Crp.T414R	this study	MP16-20
Clone 12	selected clone of Pop 12; ArcA.R120H; CpdA.G202S	this study	MP16-40
**Strains derived from co-evolved clones**			
Clone 1	plasmid-free segregant of Clone 1_{VIM}	this study	MP16-21
Clone 2	plasmid-free segregant of Clone 2_{VIM}	this study	MP16-22
Clone 3	plasmid-free segregant of Clone 3_{VIM}	this study	MP16-23
Clone 4	plasmid-free segregant of Clone 4_{VIM}	this study	MP16-24
Clone 2+VIM	Clone 2 transformed with ancestral pG06-VIM-1	this study	MP16-25
Clone 3+VIM	Clone 3 transformed with ancestral pG06-VIM-1	this study	MP16-26

Clone 2+NDM	Clone 2 transconjugant harbouring pK71-77-1-NDM	this study	MP16-27
Clone 3+NDM	Clone 3 transconjugant harbouring pK71-77-1-NDM	this study	MP16-28
Clone 12+VIM	Clone 12 transformed with ancestral pG06-VIM-1	this study	MP16-41
ExPEC+evVIM	ExPEC transformed with evolved pG06-VIM-1 isolated from Clone 2_{VIM}	this study	MP16-42
**Deletion strains**			
BW25113	*E. coli* K-12 BW25113; parent strain of the Keio collection	[29]	MP16-31
BWΔ*cpdA*	*E. coli* K-12 BW25113, Δ*cpdA*729::kan; Keio collection JW3000-1	[1]	MP16-32
BWΔ*arcA*	*E. coli* K-12 BW25113, Δ*arcA*726::kan; Keio collection JW4364-1	[1]	MP16-33
BWΔ*crp*	*E. coli* K-12 BW25113, Δ*crp*-765::kan; Keio collection JW5702-2	[1]	MP16-34
BW25113+VIM	BW25113 transformed with ancestral pG06-VIM-1	this study	MP16-35
BWΔ*cpdA*+VIM	BW25113 transformed with ancestral pG06-VIM-1	this study	MP16-36
BWΔ*arcA*+VIM	BW25113 transformed with ancestral pG06-VIM-1	this study	MP16-37
BWΔ*crp*+VIM	BW25113 transformed with ancestral pG06-VIM-1	this study	MP16-38
**Strains used in conjugation experiments**			
K56-43 RifR	spontaneous rifampicin-resistant mutant of *E. coli* K56-43	[4]	MP16-29
MG1655 Δ*ara* RifR	*E. coli* MG1655; Δ*ara*; rifampicin-resistant	[5]	MP03-11
MG1655 Δ*ara* RifR+NDM	*E. coli* MG1655 Δ*ara*; rifampicin-resistant transconjugant harbouring pK71-77-1-NDM; counterselectable pK71-77-1-NDM donor	this study	MP16-30
K71-77	*E. coli*; donor of ancestral pK71-77-1-NDM; contains also pK71-77-2	[4]	MP08-04

[a] plasmid/strain/population name in main article
[b] strain identifier at NCBI Sequence Read Archive, BioProject accession PRJNA630076

Supplementary Table 2. Primers used in this study.

Target	Direction	Sequence (5'-3')	Comment	Source or reference
repB	F	TCGCTTCATTCCTGCTTCAGC	IncR replicon; characterization of pG06-VIM-1-transformants	[30]
	R	GTGTGCTGTGGTTATGCCTCA		
bla$_{NDM-1}$	F	CAGCAAATGGAAACTGGCGACCAA	carbapenemase gene; characterization of pK71-77-1-NDM-transconjugants	in-house
	R	ACGGTGATATTGTCACTGGTGTGG		
iterons and *repE*	F	CCATGCTGGTTCTAGAGAAGGTG	IncFIA replicon (pK71-77-2); characterization of pK71-77-1-NDM-transconjugants	[31]
	R	GTATATCCTTACTGGCTTCCGCAG		
adk	F	ATTCTGCTTGGCGCTCCGGG	*E. coli* house-keeping gene; confirmation absence of genomic DNA during RNA isolation	[32]
	R	CCGTCAACTTTCGCGTATTT		
arcA	F	TGGAAAGTGCATCAAGAACG	mutational target gene; verification of identified mutations; verification of Δ*arcA*	this study
	R	TTCACTGCCGAAAATGAAAG		
arcB	F_1	GCAGGTTGTCGTGAAGGAAT	mutational target gene; verification of identified mutations	this study
	R_1	TTACCGCCATGACTGTCTTTC		
	F_2	GCTGTCACGTTGGGGAATA		
	R_2	GTCTAGCCGGGGTCATTTTT		
cpdA	F	GAAGTGTGTTCAAGCCAGCA	mutational target gene; verification of identified mutations; verification of Δ*cpdA*	this study
	R_1	CCAGTTTACGTTCCAGCCAC		
	R_2	TGACGAACCGACAATACCCA		
crp	F	AGCATATTTCGGCAATCCAG	mutational target gene; verification of identified mutations; verification of Δ*crp*	this study
	R	TAAATCAGTCTGCGCCACAT		
yncE	F	GCATTGTGAAGCAGCAAAAA	verification of identified mutation in Clone 4_{VIM}	this study
	R	CGCGTCAATCACACTGACTT		

Supplementary Table 3. Annotated genes present in deleted regions of evolved pK71-77-1-NDM.

Position	Gene/locus tag	Product	Deleted in
27576..28838	FHY70_00185	IS*1380*-like element IS*Ec9* family transposase	Clone 7_{NDM}
29162..30307	FHY70_00190	class C beta-lactamase CMY-6	Clone 7_{NDM}
30401..30934	FHY70_00195	lipocalin family protein	Clone 7_{NDM}
30931..31248	FHY70_00200	quaternary ammonium compound-resistance protein SugE	Clone 7_{NDM}
31505..31930	FHY70_00205	LuxR family transcriptional regulator	Clone 7_{NDM}
31976..37462	FHY70_00210	DUF4165 domain-containing protein	Clone 7_{NDM}
37611..38318	FHY70_00215	DsbC family protein	Clone 7_{NDM}
38315..40762	*traC*	type IV secretion system protein TraC	Clone 7_{NDM}
40777..41094	FHY70_00225	hypothetical protein	Clone 7_{NDM}
41091..41621	FHY70_00230	S26 family signal peptidase	Clone 7_{NDM}
41584..42849	FHY70_00235	conjugal transfer protein TraW	Clone 7_{NDM}
42846..43517	FHY70_00240	EAL domain-containing protein	Clone 7_{NDM}
43514..44521	FHY70_00245	conjugal transfer protein TraU	Clone 7_{NDM}
44625..47432	*traN*	conjugal transfer mating pair stabilization protein TraN	Clone 7_{NDM}
47471..48331	FHY70_00255	hypothetical protein	Clone 7_{NDM}
48454..49095	FHY70_00260	hypothetical protein	Clone 7_{NDM}
49390..49710	FHY70_00265	hypothetical protein	Clone 7_{NDM}
50014..50199	FHY70_00270	hypothetical protein	Clone 7_{NDM}
50419..51387	FHY70_00275	CbbQ/NirQ/NorQ/GpvN family protein	Clone 7_{NDM}
51398..52306	FHY70_00280	hypothetical protein	Clone 7_{NDM}
52367..52897	*ssb*	single-stranded DNA-binding protein	Clone 7_{NDM}
52992..53981	*bet*	phage recombination protein Bet	Clone 7_{NDM}
54044..55054	FHY70_00295	endonuclease	Clone 7_{NDM}
54960..55172	FHY70_00300	hypothetical protein	Clone 7_{NDM}
55254..56531	FHY70_00305	DUF3150 domain-containing protein	Clone 7_{NDM}
56616..58412	FHY70_00310	VWA domain-containing protein	Clone 7_{NDM}
58474..58809	FHY70_00315	hypothetical protein	Clone 7_{NDM}
59074..59508	FHY70_00320	hypothetical protein	Clone 7_{NDM}
59563..59766	FHY70_00325	hypothetical protein	Clone 7_{NDM}
59838..60443	FHY70_00330	5'-deoxynucleotidase	Clone 7_{NDM}
60436..60705	FHY70_00335	hypothetical protein	Clone 7_{NDM}
60719..60937	FHY70_00340	hypothetical protein	Clone 7_{NDM}
61011..61568	FHY70_00345	pyruvate dehydrogenase	Clone 7_{NDM}
61643..62494	FHY70_00350	NgrC	Clone 7_{NDM}
62953..63339	FHY70_00355	hypothetical protein	Clone 7_{NDM}
63517..65244	FHY70_00360	DNA primase	Clone 7_{NDM}
65231..65509	FHY70_00365	hypothetical protein	Clone 7_{NDM}
65582..65803	FHY70_00370	hypothetical protein	Clone 7_{NDM}
65985..66989	FHY70_00375	IS*110*-like element IS*4321* family transposase	Clone 7_{NDM}
67068..70040	FHY70_00380	Tn*3* family transposase	Clone 7_{NDM}
70043..70600	FHY70_00385	recombinase family protein	Clone 7_{NDM}
70638..70961	FHY70_00390	transposase	Clone 7_{NDM}

70906..71919	*intI1*	class 1 integron integrase IntI1	Clone 7_{NDM}
72126..72704	*aac(6')-Ib*	AAC(6')-Ib family aminoglycoside 6'-N-acetyltransferase	Clone 7_{NDM}
72873..73220	FHY70_00405	quaternary ammonium compound efflux SMR transporter QacE delta 1	Clone 7_{NDM}
73214..74053	*sul1*	sulfonamide-resistant dihydropteroate synthase Sul1	Clone 7_{NDM}
74180..74422	FHY70_00415	hypothetical protein	Clone 7_{NDM}
74506..74805	FHY70_00420	DUF2293 domain-containing protein	Clone 7_{NDM}
74919..75089	FHY70_00425	CopG family transcriptional regulator	Clone 7_{NDM}
75422..75973	FHY70_00430	DNA cytosine methyltransferase	Clone 7_{NDM}
76016..76210	FHY70_00435	hypothetical protein	Clone 7_{NDM}
76316..76969	FHY70_00440	endonuclease III	Clone 7_{NDM}
77067..77912	*rmtC*	RmtC family 16S rRNA (guanine(1405)-N(7))-methyltransferase	Pop 5 $_{NDM}$<br>Clone 5_{NDM}<br>Clone 7_{NDM}
78222..78919	FHY70_00450	IS*1* family transposase	Pop 5 $_{NDM}$<br>Clone 5_{NDM}<br>Clone 7_{NDM}
78925..79071	FHY70_00455	transposase	Pop 5 $_{NDM}$<br>Clone 5_{NDM}<br>Clone 7_{NDM}
79204..80064	FHY70_00460	aminoglycoside N-acetyltransferase AAC(3)-IId	Pop 5 $_{NDM}$<br>Clone 5_{NDM}<br>Clone 7_{NDM}
80077..80619	FHY70_00465	tunicamycin resistance protein	Pop 5 $_{NDM}$<br>Clone 5_{NDM}<br>Clone 7_{NDM}
81101..81292	FHY70_00470	hypothetical protein	Pop 5 $_{NDM}$<br>Clone 5_{NDM}<br>Clone 7_{NDM}
81298..81543	FHY70_00475	hypothetical protein	Pop 5 $_{NDM}$<br>Clone 5_{NDM}<br>Clone 7_{NDM}
81594..82730	FHY70_00480	DUF3883 domain-containing protein	Pop 5 $_{NDM}$<br>Clone 5_{NDM}<br>Clone 7_{NDM}
82845..84216	FHY70_00485	IS*1182*-like element IS*Cfr1* family transposase	Pop 5 $_{NDM}$<br>Clone 5_{NDM}<br>Clone 7_{NDM}
84816..85235	FHY70_00490	ATP-binding protein	Pop 5 $_{NDM}$<br>Clone 5_{NDM}<br>Clone 7_{NDM}
85238..85672	FHY70_00495	hypothetical protein	Pop 5 $_{NDM}$<br>Clone 5_{NDM}<br>Clone 7_{NDM}
85658..85780	FHY70_00500	metal-dependent hydrolase	Pop 5 $_{NDM}$<br>Clone 5_{NDM}<br>Clone 7_{NDM}
85726..85845	FHY70_00505	IS*6* family transposase	Pop 5 $_{NDM}$<br>Clone 5_{NDM}<br>Clone 7_{NDM}
85901..86598	FHY70_00510	IS*1* family transposase	Pop 5 $_{NDM}$<br>Clone 5_{NDM}<br>Clone 7_{NDM}

Supplementary Table 4. Mutations identified in evolved populations[a, b].

Gene	Position	Mutation	Annotation	Pop 1$_{VIM}$	Pop 2$_{VIM}$	Pop 3$_{VIM}$	Pop 4$_{VIM}$	Pop 5$_{NDM}$	Pop 6$_{NDM}$	Pop 7$_{NDM}$	Pop 8$_{NDM}$	Pop 9	Pop 10	Pop 11	Pop 12
arcA	4289006	G→A	G36D (GGC→GAC)						2,2						
	4289014	A→G	M39V (ATG→GTG) ‡	15,2											
	4289016	G→A	M39I (ATG→ATA) ‡		1,2				15,6			2,2			
	4289048	T→A	L50Q (CTG→CAG)			100	7,2			1,2		1,2	1,7		
	4289058	G→A	M53I (ATG→ATA)							38,9	33,5				
	4289068	C→A	L57M (CTG→ATG)											2,6	
	4289079	A→C	K60N (AAA→AAC)										93		
	4289126	C→A	A76E (GCG→GAG)					4,5							
	4289134	T→C	F79L (TTC→CTC) ‡		31,6							33,2			
	4289135	T→A	F79Y (TTC→TAC) ‡				91,4		2,6			17,1	1,1		
	4289168	T→C	I90T (ATT→ACT)									8,1			
	4289173	G→A	G92S (GGC→AGC)						9,2						
	4289176	C→T	L93F (CTC→TTC)					94,5							
	4289179	G→A	E94K (GAA→AAA)	81											
	4289210	C→A	P104Q (CCG→CAG)								1,5		3,4		
	4289212	T→C	F105L (TTC→CTC)								19,4	17,5			
	4289234	T→G	I112S (ATT→AGT) ‡											4,9	
	4289235	T→G	I112M (ATT→ATG) ‡								12,6				
	4289243	G→T	R115L (CGC→CTC)						3,8		30,6				
	4289249	T→A	L117Q (CTG→CAG)		59,5									11,8	
	4289252	T→A	L118Q (CTG→CAG)		4,3				1,5						
	4289258	G→A	R120H (CGT→CAT)											75,1	100
	4289357	G→T	G153V (GGC→GTC)											5,1	
	4289384	C→T	P162L (CCG→CTG)						3						
	4289512	A→C	I205L (ATC→CTC)						2,2			2,6			
arcB	559547	T→G	Y11D (TAT→GAT)						1,5						
	559992	C→T	S159F (TCC→TTC)									5,3			
	560321	C→T	R269C (CGT→TGT)								2,1				
	560356	C→A	S280R (AGC→AGA)								1,2	3,7			
	560744	G→A	A410T (GCC→ACC)						2,2						
	560754	T→G	F413C (TTC→TGC)									3,4			
	560841	T→C	I442T (ATT→ACT)							47,1					
	561252	T→G	L579W (TTG→TGG)						27						
	561574	A→C	L686F (TTA→TTC)						22,3						
	561581	T→G	F689V (TTT→GTT)							3,1					
	561717	T→G	L734R (CTG→CGG)							7,2					
	561754	T→G	C746W (TGT→TGG)									2,4			
cpdA	731329	A→C	D22A (GAC→GCC)												4,1
	731334	C→T	H24Y (CAC→TAC) ‡									3,5			
	731335	A→G	H24R (CAC→CGC) ‡						1,7						
	731385	A→C	S41R (AGC→CGC)									3,3			
	731533	Δ1 bp	coding (269/828 nt)			8,5									
	731704	Δ4 bp	coding (440-443/828 nt)											1,1	
	731742	+TGC	coding (478/828 nt)	7,5	5,6				15,3					8,9	
	731743	Δ3 bp	coding (488-490/828 nt)		64			100	25,4	2,1	38,1	48	1,7		
	731754	Δ3 bp	coding (490-492/828 nt)									2,3			
	731757	C→T	H165Y (CAT→TAT)							1,2				1,6	
	731778	Δ13 bp	coding (514-526/828 nt)								12,5				
	731784	T→G	W174G (TGG→GGG)						4,3						
	731805	+GTAA	coding (541/828 nt)				74,7								
	731833	T→A	L190Q (CTG→CAG)										1,8		
	731863	T→G	L200R (CTG→CGG)									3,1			
	731868	G→A	G202S (GGT→AGT)												70,2
	731869	+TCACAT	coding (605/828 nt)						3,5						
	731871	C→T	H203Y (CAC→TAC)								12,5				
	731877	C→T	H205Y (CAT→TAT)								1,1				
	731925	C→A	P221T (CCG→ACG) ‡								1,2				
	731926	C→T	P221L (CCG→CTG) ‡				9,3								
	731929	C→T	S222L (TCG→TTG)	13,6											
	732027	A→C	T255P (ACC→CCC)											32,9	
crp	427694	A→C	L196R (CTG→CGG)										95,8		
	427712	A→C	M190R (ATG→AGG)												19,6
	427835	A→C	L149R (CTG→CGG)											4,1	
	427847	G→A	A145V (GCA→GTA) ‡			9,9								7,7	
	427848	C→T	A145T (GCA→ACA) ‡								4,2	34,1			1,5
	427857	C→T	G142S (GGC→AGC)	81,8		77,3	7,5		60,7	94,1					
	427859	G→C	T141R (ACG→AGG)								33,2				
	427878	G→T	L135M (CTG→ATG)											26,9	
cyaA ← / → hemC	5034928	G→T	intergenic (-9/-378)		34,7										
cyaA	5032557	G→A	S788L (TCG→TTG)		1,6										
polA	4951215	+GCTGCACGTTCAGCC	coding (2529/2787 nt)									19,9			
yncE	2595778	G→A	T178T (ACC→ACT)				100								

[a] ‡ = point mutations targeting the same codon

[b] grey background filling = mutations that were found in more than one evolved population

Supplementary Table 5. Descriptive statistics from clonal and population short-read whole-genome sequencing data alignment to sequences of ancestral reference strain ExPEC and the respective plasmid.

Clone/Population	Read number	Mean coverage chromosome	Mean coverage plasmid[a]	Plasmid copy number[a]
ExPEC	4196177	113	n/a	n/a
ExPEC+VIM	3242939	91	118	1,3
ExPEC+NDM	7187517	200	265	1,3
Clone 1_{VIM}	4778901	141	123	0,9
Clone 2_{VIM}	1715202	48	70	1,5
Clone 3_{VIM}	6000473	159	137	0,9
Clone 4_{VIM}	8490236	229	243	1,1
Clone 5_{NDM}	3731727	99	146	1,5
Clone 6_{NDM}	3451700	97	141	1,5
Clone 7_{NDM}	2486214	66	94	1,4
Clone 8_{NDM}	2539041	69	82	1,2
Clone 2	3535346	98	n/a	n/a
Clone 3	4156737	117	n/a	n/a
**average**	**4149187**	**117×**	**142×**	

Pop 1_{VIM}	24207523	650	1432
Pop 2_{VIM}	51031466	1412	2063
Pop 3_{VIM}	18387700	509	1132
Pop 4_{VIM}	22787328	644	363
Pop 5_{NDM}	57493948	1590	2428
Pop 6_{NDM}	37134452	1031	1446
Pop 7_{NDM}	58178599	1702	2217
Pop 8_{NDM}	78036459	2141	3322
Pop 9	57282733	1611	n/a
Pop 10	62460521	1894	n/a
Pop 11	25260332	738	n/a
Pop 12	63990083	1938	n/a
**average**	**43407184**	**1322×**	**1800×**

[a] n/a = not applicable

Supplementary Table 6. Summary of the results from linear regression analysis (serial competition experiments).

Competitors	n	Mean relative fitness (*w*)[a]	SD[b] *w*	Shapiro–Wilk test	Min/max *w*		Mean selection coefficient (*s*)[c]	*P s*	Significance *s*[d]	*P* Dunnet[e]	Significance Dunnet[e]
ExPEC vs ExPEC+VIM	4	0.947	0.002	0.766	0.944	0.950	-0.053	2.38×10^{-5}	***	control group	control group
Clone 1 vs Clone 1$_{VIM}$	3	0.993	0.002	0.993	0.991	0.994	-0.007	0.017	*	$< 10^{-10}$	***
Clone 2 vs Clone 2$_{VIM}$	3	0.996	0.001	0.336	0.995	0.997	-0.004	0.016	*	$< 10^{-10}$	***
Clone 3 vs Clone 3$_{VIM}$	3	0.991	0.0004	0.122	0.990	0.991	-0.009	0.001	***	$< 10^{-10}$	***
Clone 4 vs Clone 4$_{VIM}$	3	0.994	0.002	0.271	0.992	0.996	-0.006	0.056	ns	$< 10^{-10}$	***
Clone 2 vs Clone 2+VIM	3	0.987	0.004	0.290	0.985	0.992	-0.013	0.026	*	2.51×10^{-7}	***
Clone 3 vs Clone 3+VIM	3	0.990	0.001	0.365	0.989	0.990	-0.010	0.002	**	1.78×10^{-7}	***
Clone 12 vs Clone 12+VIM	4	0.991	0.002	0.410	0.988	0.994	-0.009	0.009	**	n/a[f]	n/a
ExPEC vs ExPEC+evVIM	3	0.968	0.005	0.376	0.963	0.974	-0.032	0.01	**	n/a	n/a
ExPEC vs ExPEC+NDM	3	0.945	0.012	0.627	0.932	0.956	-0.055	0.017	*	control group	control group
Clone 2 vs Clone 2+NDM	3	0.976	0.007	0.553	0.971	0.984	-0.024	0.025	*	0.006	**
Clone 3 vs Clone 3+NDM	3	0.973	0.002	0.489	0.972	0.976	-0.027	0.002	**	0.010	*
BW25113 vs BW25113+VIM	3	0.977	0.005	0.238	0.974	0.983	-0.023	0.016	*	control group	control group
BW∆*arcA* vs BW∆*arcA*+VIM	4	0.978	0.007	0.436	0.972	0.988	-0.022	0.007	**	0.993	ns
BW∆*cpdA* vs BW∆*cpdA*+VIM	4	0.996	0.002	0.750	0.994	0.999	-0.004	0.034	*	0.0008	***
BW∆*crp* vs BW∆*crp*+VIM	5	1.006	0.020	0.640	0.977	1.026	0.006	0.534	ns	0.051	ns

[a] relative fitness $w = 1+s$, where the fitness of the plasmid-free strain equals 1
[b] SD = standard deviation
[c] selection coefficient $s = 0.5 * b/\ln(1/d)$ with *b* (= slope) obtained from regressing the natural logarithm of the ratio ($CFU_{plasmid\text{-}carrying}/CFU_{plasmid\text{-}free}$) over timepoints (= T_0-T_{72}) and *d* as the dilution factor at each timepoint (here 1:100)
[d] one-sample *t*-test, two-sided; significance levels are indicated by asterisks (*P* = * < 0.05, ** < 0.01, *** < 0.001; ns = not significant)
[e] Dunnett's multiple comparison performed relative to the strain/strain-plasmid combination indicated as control group; significance levels are indicated by asterisks (*P* = * < 0.05, ** < 0.01, *** < 0.001; ns = not significant)
[f] not applicable

Supplementary Table 7. Summary of the results from linear regression analysis (plasmid loss).

Strain	n	Mean slope (*b*)[a]	SD[b] *b*	*P b*	Significance *b*[c]	Mean frequency at T_{72}	SD frequency at T_{72}	*P* frequency at T_{72}	Significance frequency[c]	Plasmid stability[d]
ExPEC+VIM	3	-0.011	0.002	0.017	*	0.967	0.065	0.477	ns	stable
Clone 1_{VIM}	3	-0.012	0.026	0.501	ns	0.877	0.147	0.284	ns	stable
Clone 2_{VIM}	3	0.001	0.026	0.957	ns	0.849	0.052	0.037	*	stable
Clone 3_{VIM}	3	-0.009	0.012	0.345	ns	0.790	0.048	0.017	*	stable
Clone 4_{VIM}	3	-0.017	0.019	0.264	ns	0.892	0.116	0.248	ns	stable
ExPEC+NDM	3	-0.006	0.011	0.458	ns	0.971	0.071	0.559	ns	stable
Clone 2+VIM	3	-0.017	0.006	0.036	*	0.860	0.056	0.049	*	unstable[e]
Clone 3+VIM	3	-0.004	0.027	0.824	ns	0.916	0.083	0.219	ns	stable
Clone 12+VIM	4	-0.024	0.018	0.076	ns	0.915	0.064	0.077	ns	stable
ExPEC+evVIM	3	-0.020	0.022	0.252	ns	0.882	0.034	0.027	*	stable
Clone 2+NDM	3	0.016	0.009	0.098	ns	0.998	0.027	0.893	ns	stable
Clone 3+NDM	3	-0.009	0.016	0.459	ns	0.944	0.077	0.330	ns	stable
BW25113+VIM	3	-0.015	0.059	0.702	ns	0.802	0.085	0.056	ns	stable
BWΔarcA+VIM	3	0.040	0.042	0.247	ns	0.998	0.143	0.981	ns	stable
BWΔcpdA+VIM	3	0.005	0.036	0.848	ns	1.103	0.113	0.254	ns	stable
BWΔcrp+VIM	3	-0.012	0.042	0.665	ns	0.981	0.189	0.878	ns	stable

[a] slope obtained by regressing the frequency of the plasmid-carrying population ($CFU_{plasmid\text{-}carrying}$/ CFU_{total}) over timepoint (= T_0-T_{72})
[b] SD = standard deviation
[c] one-sample *t*-test, two-sided; significance levels are indicated by asterisks (*P* = * < 0.05, ** < 0.01, *** < 0.001; ns = not significant)
[d] plasmids were considered stable if a *P*-value for either '*b*' or 'Mean frequency at T_{72}' was non-significant
[e] in Clone 2+VIM, 'Mean frequency at T_{72}' is only borderline significant

Supplementary Table 8. Results from susceptibility testing of ancestral and evolved pK71-77-1-NDM-carrying clones by disc diffusion test[a,b].

	**Tobramycin (10 µg)**	**Gentamicin (10 µg)**
ATCC 25922[c]	18 = S	19 = S
	19 = S	19 = S
	19 = S	19 = S
	**19 = S**	**19 = S**
Clone 5_{NDM}	8 = R	17 = S
	8 = R	18 = S
	7 = R	18 = S
	**8 = R**	**18 = S**
Clone 6_{NDM}	no inhibition zone = R	no inhibition zone = R
	no inhibition zone = R	no inhibition zone = R
	no inhibition zone = R	no inhibition zone = R
	no inhibition zone = **R**	no inhibition zone = **R**
Clone 7_{NDM}	20 = S	20 = S
	20 = S	20 = S
	20 = S	21 = S
	**20 = S**	**20 = S**
Clone 8_{NDM}	no inhibition zone = R	no inhibition zone = R
	no inhibition zone = R	no inhibition zone = R
	no inhibition zone = R	no inhibition zone = R
	no inhibition zone = **R**	no inhibition zone = **R**
ExPEC+NDM	no inhibition zone = R	no inhibition zone = R
	no inhibition zone = R	no inhibition zone = R
	no inhibition zone = R	no inhibition zone = R
	no inhibition zone = **R**	no inhibition zone = **R**

[a] S = susceptible; R = resistant (according to [20])
[b] median inhibition zone diameter (mm)
[c] control strain

Supplementary Table 9. Effect of non-synonymous mutations on protein function in evolved populations[a].

	PROVEAN[b]		SIFT[c]		SNAP2[d]			overall effect[e]
	score	effect	score	effect	score	effect	expected accuracy	
arcA								
**G36D**	-5.33	Deleterious	0.01	AFFECT PROTEIN FUNCTION	79	effect	85%	3/3
**M39V**	-2.95	Deleterious	-	TOLERATED	15	effect	59%	2/3
**M39I**	-2.916	Deleterious	0.32	TOLERATED	28	effect	65%	2/3
**L50Q**	-5.513	Deleterious	0	AFFECT PROTEIN FUNCTION	45	effect	71%	3/3
**M53I**	-0.87	Neutral	0.06	TOLERATED	32	effect	66%	3/3
**L57M**	-1.631	Neutral	0	AFFECT PROTEIN FUNCTION	-93	neutral	97%	2/3
**K60N**	-2.679	Deleterious	0.14	TOLERATED	45	effect	71%	2/3
**A76E**	-2.102	Neutral	0.09	TOLERATED	70	effect	85%	2/3
**F79L**	0.765	Neutral	-	TOLERATED	-47	neutral	72%	3/3
**F79Y**	-1.549	Neutral	0.03	AFFECT PROTEIN FUNCTION	60	effect	80%	2/3
**I90T**	-4.928	Deleterious	0.01	AFFECT PROTEIN FUNCTION	50	effect	75%	3/3
**G92S**	-4.827	Deleterious	0	AFFECT PROTEIN FUNCTION	74	effect	85%	3/3
**L93F**	-3.93	Deleterious	0	AFFECT PROTEIN FUNCTION	1	effect	53%	3/3
**E94K**	-3.996	Deleterious	0	AFFECT PROTEIN FUNCTION	80	effect	91%	3/3
**P104Q**	-7.993	Deleterious	0	AFFECT PROTEIN FUNCTION	80	effect	91%	3/3
**F105L**	-4.943	Deleterious	0.41	TOLERATED	44	effect	71%	2/3
**I112M**	-1.414	Neutral	0.09	TOLERATED	-63	neutral	82%	3/3
**I112S**	-2.444	Neutral	0.06	TOLERATED	-26	neutral	61%	3/3
**R115L**	-5.594	Deleterious	0.01	AFFECT PROTEIN FUNCTION	61	effect	80%	3/3
**L117Q**	-5.665	Deleterious	0.01	AFFECT PROTEIN FUNCTION	13	effect	59%	3/3
**L118Q**	-4.794	Deleterious	0	AFFECT PROTEIN FUNCTION	21	effect	63%	3/3
**R120H**	-4.837	Deleterious	0	AFFECT PROTEIN FUNCTION	90	effect	95%	3/3
**G153V**	-1.529	Neutral	0.25	TOLERATED	-22	neutral	61%	3/3
**P162L**	-3.961	Deleterious	0.06	TOLERATED	61	effect	80%	2/3
**I205L**	-1.88	Neutral	0	AFFECT PROTEIN FUNCTION	-2	neutral	53%	2/3
arcB								
**Y11D**	-6.971	Deleterious	0	AFFECT PROTEIN FUNCTION	79	effect	85%	3/3
**S159F**	-5.335	Deleterious	0.02	AFFECT PROTEIN FUNCTION	24	effect	63%	3/3
**R269C**	-6.825	Deleterious	0.01	AFFECT PROTEIN FUNCTION	-18	neutral	57%	2/3
**S280R**	-4.062	Deleterious	0.04	AFFECT PROTEIN FUNCTION	21	effect	63%	3/3
**A410T**	-3.762	Deleterious	0.01	AFFECT PROTEIN FUNCTION	49	effect	71%	3/3
**F413C**	-7.599	Deleterious	0	AFFECT PROTEIN FUNCTION	20	effect	63%	3/3
**I442T**	-4.779	Deleterious	0.03	AFFECT PROTEIN FUNCTION	-4	neutral	53%	2/3
**L579W**	-5.787	Deleterious	0	AFFECT PROTEIN FUNCTION	59	effect	75%	3/3

**L686F**	-2.213	Neutral	0.13	TOLERATED	-39	neutral	66%	3/3
**F689V**	-6.474	Deleterious	0	AFFECT PROTEIN FUNCTION	33	effect	66%	3/3
**L734R**	-3.729	Deleterious	0.01	AFFECT PROTEIN FUNCTION	-17	neutral	57%	2/3
**C746W**	11.174	Neutral	0.02	AFFECT PROTEIN FUNCTION	-92	neutral	97%	2/3
cpdA								
**D22A**	-7.763	Deleterious	0	AFFECT PROTEIN FUNCTION	95	effect	95%	3/3
**H24R**	-7.765	Deleterious	-	AFFECT PROTEIN FUNCTION	94	effect	95%	3/3
**H24Y**	-5.825	Deleterious	0	AFFECT PROTEIN FUNCTION	92	effect	95%	3/3
**S41R**	-4.876	Deleterious	0.71	TOLERATED	4	effect	53%	2/3
**H165Y**	-5.893	Deleterious	0	AFFECT PROTEIN FUNCTION	81	effect	91%	3/3
**W174G**	-12.87	Deleterious	0.31	TOLERATED	82	effect	91%	2/3
**L190Q**	-5.289	Deleterious	0.02	AFFECT PROTEIN FUNCTION	-3	neutral	53%	2/3
**L200R**	-5.507	Deleterious	0	AFFECT PROTEIN FUNCTION	70	effect	85%	3/3
**G202S**	-5.939	Deleterious	0	AFFECT PROTEIN FUNCTION	86	effect	91%	3/3
**H203Y**	-5.939	Deleterious	0	AFFECT PROTEIN FUNCTION	94	effect	95%	3/3
**H205Y**	-5.939	Deleterious	0	AFFECT PROTEIN FUNCTION	92	effect	95%	3/3
**P221L**	-9.898	Deleterious	0	AFFECT PROTEIN FUNCTION	87	effect	91%	3/3
**P221T**	-7.919	Deleterious	0	AFFECT PROTEIN FUNCTION	80	effect	91%	3/3
**S222L**	-5.558	Deleterious	0.05	TOLERATED	89	effect	91%	2/3
**T255P**	-1.36	Neutral	0.2	TOLERATED	-44	neutral	72%	3/3
crp								
**L135M**	-1.827	Neutral	0	AFFECT PROTEIN FUNCTION	-52	neutral	78%	2/3
**T141R**	-4.042	Deleterious	0.11	TOLERATED	-37	neutral	66%	2/3
**G142S**	-5.223	Deleterious	0.76	TOLERATED	50	effect	75%	2/3
**A145T**	-3.522	Deleterious	-	TOLERATED	-18	neutral	57%	2/3
**A145V**	-3.573	Deleterious	1	TOLERATED	21	effect	63%	2/3
**L149R**	-4.97	Deleterious	0.03	AFFECT PROTEIN FUNCTION	42	effect	71%	3/3
**M190R**	-0.079	Neutral	0.47	TOLERATED	-49	neutral	72%	3/3
**L196R**	-5.087	Deleterious	0	AFFECT PROTEIN FUNCTION	67	effect	80%	3/3

[a] frame-shift mutations were not investigated here
[b] PROVEAN [22]: variants with a score equal to or below -2.5 are considered "deleterious"; variants with a score above -2.5 are considered "neutral"
[c] SIFT [23]: amino acids with probabilities < 0.05 are predicted to be deleterious
[d] SNAP2 [24]: values range from -100 (neutral) to 100 (effect)
[e] red: deleterious effect on protein function; green: neutral effect on protein function

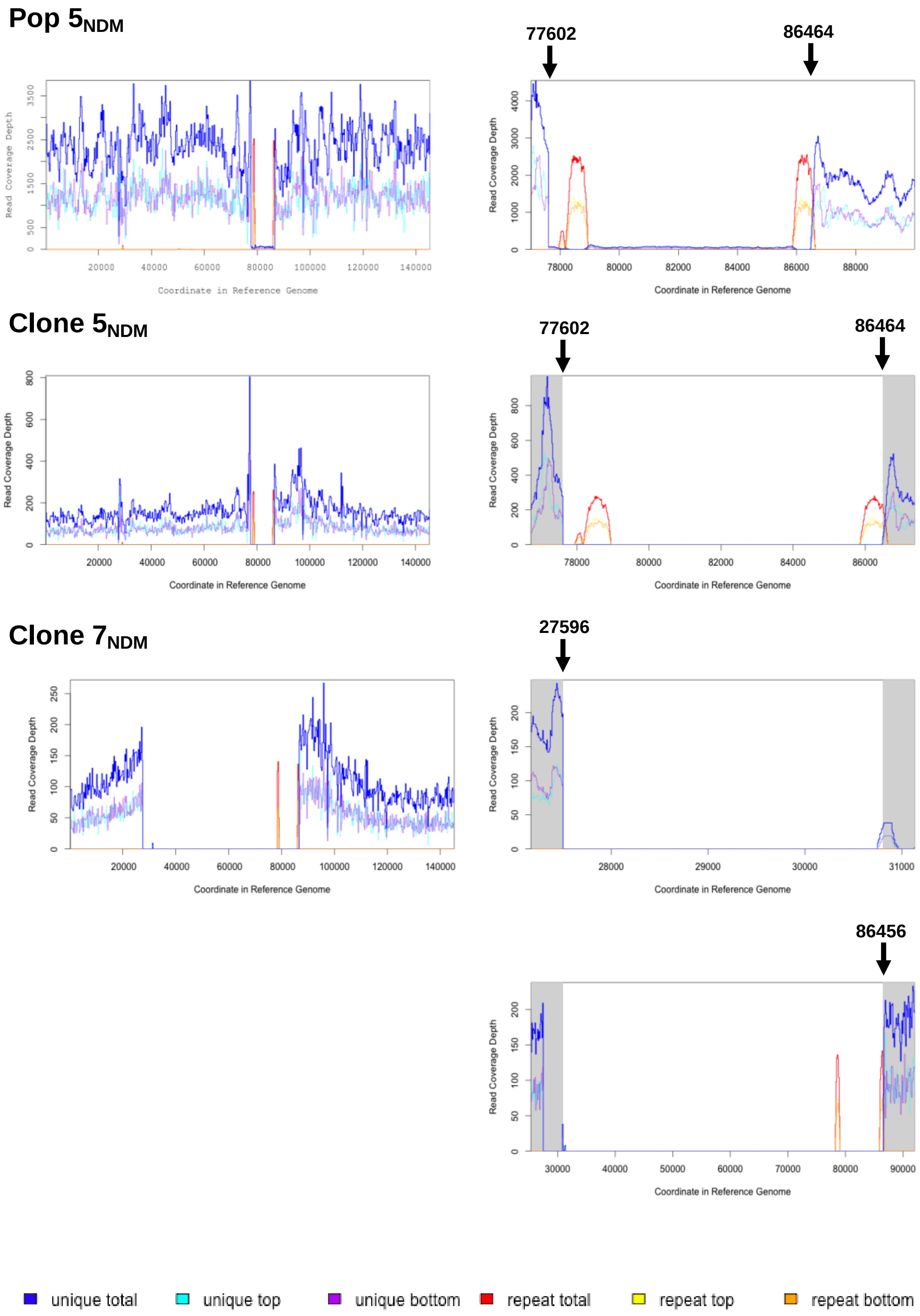

Supplementary Figure 1. Read coverage plots of evolved pK71-77-1-NDM in evolved Pop 5_{NDM}, Clone 5_{NDM} and Clone 7_{NDM}. Large deletions in the evolved plasmid sequences were identified when mapping short-read sequencing data against the ancestral pK71-77-1-NDM using breseq [10] (see also main text Methods). Plots were part of the breseq output. The read coverage depth across the whole plasmid (145 kb; left column) and around specified areas with missing coverage (white background; right column) is shown. Beginning and end positions of the largest possible deletion (including repetitive regions indicated in red/yellow/orange) are displayed and were accessed using the bam2cov command in breseq. A colour legend common to all plots is given at the bottom.

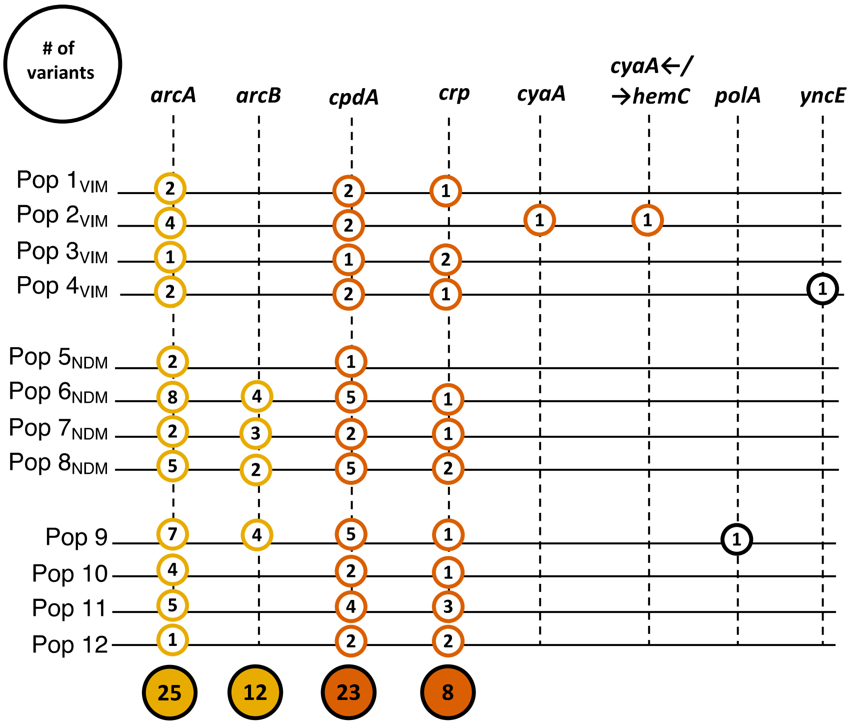

Supplementary Figure 2. Number of chromosomal variants per evolved population and mutational target gene. Total number of unique variants per gene are summarized in filled circles.

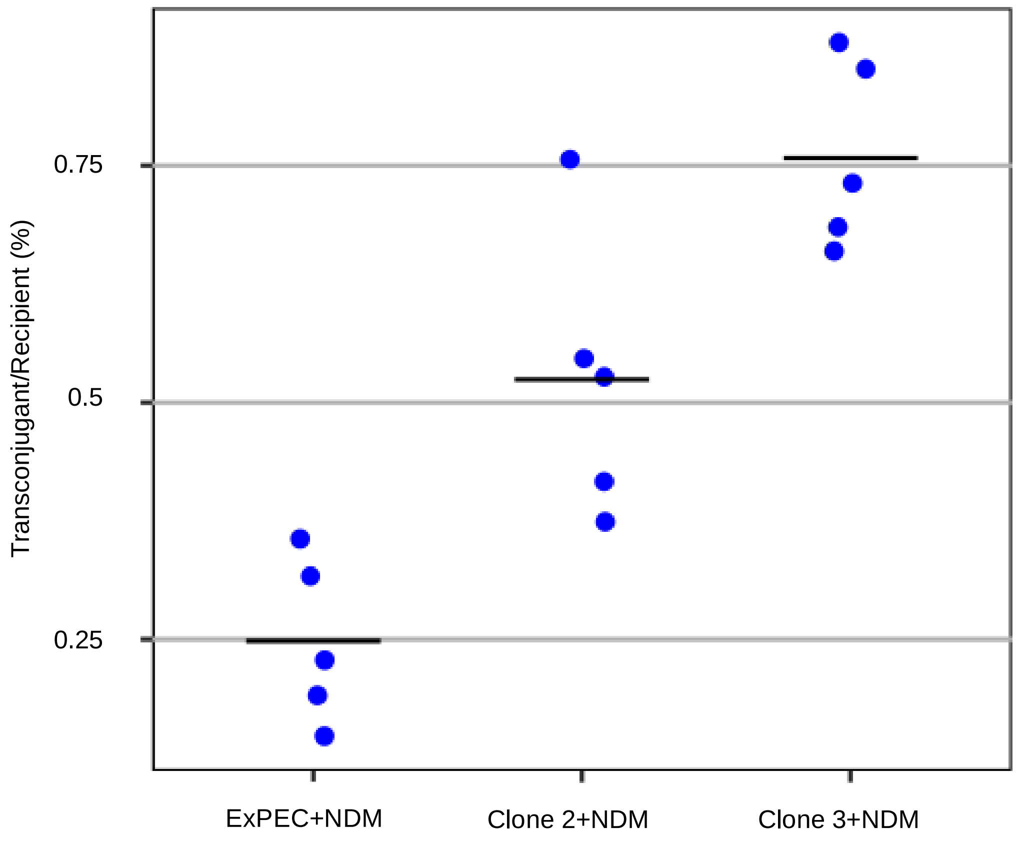

Supplementary Figure 3. Conjugation frequency of ancestral pK71-77-1-NDM from ancestral and evolved backgrounds (ExPEC+NDM and Clone 2+NDM/Clone 3+NDM, respectively) into strain K56-43 RifR. The plasmid was transferred with an average frequency of 0.25 ± 0.08% from strain ExPEC+NDM, 0.52 ± 0.15% from Clone 2+NDM and 0.75 ± 0.09% from Clone 3+NDM (all n = 5).

Supplementary Figures 4 and 5. Ontology graphs of enrichment and overrepresentation analysis RNA-Seq. Up and downregulated processes (Supp.Figure 4 and 5, respectively) in Clone 2 and 3 with and without pG06-VIM-1 due to adaptive chromosomal mutations. Border colour indicates significant changes for: Clone 2+VIM vs. ExPEC+VIM (dark blue); Clone 3+VIM vs. ExPEC+VIM (red); both Clone 2+VIM and 3+VIM vs. ExPEC+VIM (yellow). Fill colour indicates significant changes for: Clone 2 vs. ExPEC (light blue); Clone 3 vs. ExPEC (purple); both Clone 2 and 3 vs. ExPEC (grey). The graph is read from the bottom, such that a lower biological process “is a“ (black lines) or is “part of” (light blue lines) a higher connecting process.

Supplementary Figure 6. Ontology graph of enrichment and overrepresentation analysis RNA-Seq. Downregulated processes due to pG06-VIM-1 acquisition in Clone 2+VIM vs. ExPEC+VIM. Fill colour: significant changes in Clone 2+VIM (light blue). The graph is read from the bottom, such that a lower biological process “is a“ (black lines) or is “part of” (light blue lines) a higher connecting process.

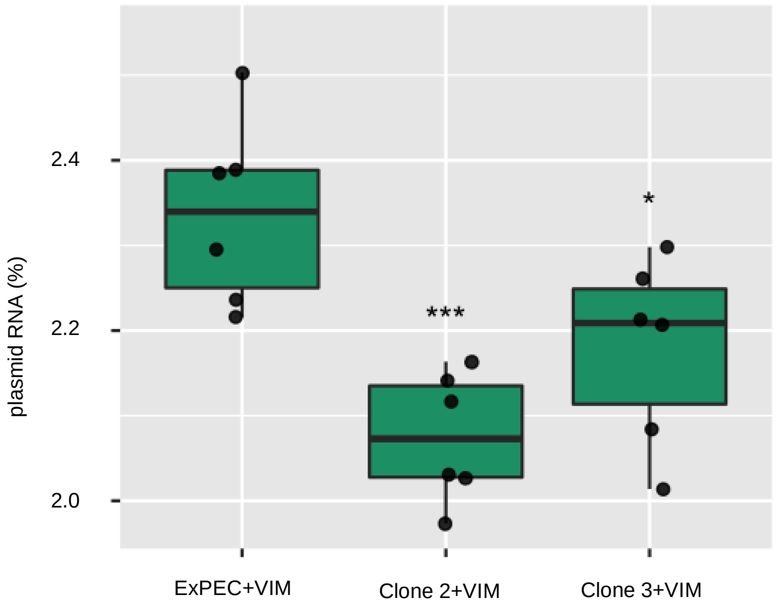

Supplementary Figure 7. Proportion of plasmid RNA transcripts. Normalized counts of plasmid RNA transcripts represent 2.33 ± 0.11% of all ExPEC+VIM transcripts, 2.08 ± 0.08% for Clone 2+VIM and 2.18 ± 0.11% for Clone 3+VIM (all n = 6).

References

1. Baba, T., et al., *Construction of Escherichia coli K-12 in-frame, single-gene knockout mutants: the Keio collection.* Mol Syst Biol, 2006. **2**: p. 2006 0008 DOI: 10.1038/msb4100050.
2. Tu, Q., et al., *Room temperature electrocompetent bacterial cells improve DNA transformation and recombineering efficiency.* Sci Rep, 2016. **6**: p. 24648 DOI: 10.1038/srep24648.
3. Sambrook, J. and D.W. Russel, *Molecular Cloning: A Laboratory Manual*. 2001, Cold Spring Harbor Laboratory Press: Cold Spring Harbor, N.Y.
4. Gama, J.A., et al., *Host dependent maintenance of a blaNDM-1-encoding plasmid in clinical Escherichia coli isolates.* Sci Rep, 2020. **10**(1): p. 9332 DOI: 10.1038/s41598-020-66239-8.
5. Gama, J.A., et al., *Temperate bacterial viruses as double-edged swords in bacterial warfare.* PLoS One, 2013. **8**(3): p. e59043 DOI: 10.1371/journal.pone.0059043.
6. Wick, R.R., *Filtlong-Quality filtering tool for long reads.* available at https://github.com/rrwick/Filtlong, 2017.
7. Wick, R.R., et al., *Unicycler: Resolving bacterial genome assemblies from short and long sequencing reads.* PLoS Comput Biol, 2017. **13**(6): p. e1005595 DOI: 10.1371/journal.pcbi.1005595.
8. Seemann, T., *Snippy: fast bacterial variant calling from NGS reads.* available at https://github.com/tseemann/snippy, 2015.
9. Tatusova, T., et al., *NCBI prokaryotic genome annotation pipeline.* Nucleic Acids Res, 2016. **44**(14): p. 6614-24 DOI: 10.1093/nar/gkw569.
10. Deatherage, D.E. and J.E. Barrick, *Identification of mutations in laboratory-evolved microbes from next-generation sequencing data using breseq.* Methods Mol Biol, 2014. **1151**: p. 165-88 DOI: 10.1007/978-1-4939-0554-6_12.
11. Frendorf, P.O., et al., *Mutations in the Global Transcription Factor CRP/CAP: Insights from Experimental Evolution and Deep Sequencing.* Comput Struct Biotechnol J, 2019. **17**: p. 730-736 DOI: 10.1016/j.csbj.2019.05.009.
12. Kim, J., S. Adhya, and S. Garges, *Allosteric changes in the cAMP receptor protein of Escherichia coli: hinge reorientation.* Proc Natl Acad Sci U S A, 1992. **89**(20): p. 9700-4 DOI: 10.1073/pnas.89.20.9700.
13. Youn, H., J. Koh, and G.P. Roberts, *Two-state allosteric modeling suggests protein equilibrium as an integral component for cyclic AMP (cAMP) specificity in the cAMP receptor protein of Escherichia coli.* J Bacteriol, 2008. **190**(13): p. 4532-40 DOI: 10.1128/JB.00074-08.
14. Popovych, N., et al., *Structural basis for cAMP-mediated allosteric control of the catabolite activator protein.* Proc Natl Acad Sci U S A, 2009. **106**(17): p. 6927-32 DOI: 10.1073/pnas.0900595106.
15. Harman, J.G., A. Peterkofsky, and K. Mckenney, *Arginine Substituted for Leucine at Position-195 Produces a Cyclic Amp-Independent Form of the Escherichia-Coli Cyclic-Amp Receptor Protein.* Journal of Biological Chemistry, 1988. **263**(17): p. 8072-8077.
16. Saxer, G., et al., *Mutations in global regulators lead to metabolic selection during adaptation to complex environments.* PLoS Genet, 2014. **10**(12): p. e1004872 DOI: 10.1371/journal.pgen.1004872.
17. Larsen, M.V., et al., *Multilocus sequence typing of total-genome-sequenced bacteria.* J Clin Microbiol, 2012. **50**(4): p. 1355-61 DOI: 10.1128/JCM.06094-11.

18. Carattoli, A., et al., *In silico detection and typing of plasmids using PlasmidFinder and plasmid multilocus sequence typing.* Antimicrob Agents Chemother, 2014. **58**(7): p. 3895-903 DOI: 10.1128/AAC.02412-14.
19. Bortolaia, V., et al., *ResFinder 4.0 for predictions of phenotypes from genotypes.* Journal of Antimicrobial Chemotherapy, 2020 DOI: 10.1093/jac/dkaa345.
20. *The European Committee on Antimicrobial Susceptibility Testing. Breakpoint tables for interpretation of MICs and zone diameters. Version 9.0.* available at http://www.eucast.org, 2019.
21. Porse, A., et al., *Survival and Evolution of a Large Multidrug Resistance Plasmid in New Clinical Bacterial Hosts.* Mol Biol Evol, 2016. **33**(11): p. 2860-2873 DOI: 10.1093/molbev/msw163.
22. Choi, Y. and A.P. Chan, *PROVEAN web server: a tool to predict the functional effect of amino acid substitutions and indels.* Bioinformatics, 2015. **31**(16): p. 2745-7 DOI: 10.1093/bioinformatics/btv195.
23. Sim, N.L., et al., *SIFT web server: predicting effects of amino acid substitutions on proteins.* Nucleic Acids Res, 2012. **40**(Web Server issue): p. W452-7 DOI: 10.1093/nar/gks539.
24. Hecht, M., Y. Bromberg, and B. Rost, *Better prediction of functional effects for sequence variants.* BMC Genomics, 2015. **16 Suppl 8**: p. S1 DOI: 10.1186/1471-2164-16-S8-S1.
25. Samuelsen, O., et al., *Molecular characterization of VIM-producing Klebsiella pneumoniae from Scandinavia reveals genetic relatedness with international clonal complexes encoding transferable multidrug resistance.* Clin Microbiol Infect, 2011. **17**(12): p. 1811-6 DOI: 10.1111/j.1469-0691.2011.03532.x.
26. Di Luca, M.C., et al., *Low biological cost of carbapenemase-encoding plasmids following transfer from Klebsiella pneumoniae to Escherichia coli.* J Antimicrob Chemother, 2017. **72**(1): p. 85-89 DOI: 10.1093/jac/dkw350.
27. Samuelsen, O., et al., *Identification of NDM-1-producing Enterobacteriaceae in Norway.* J Antimicrob Chemother, 2011. **66**(3): p. 670-2 DOI: 10.1093/jac/dkq483.
28. Kahlmeter, G. and H.O. Poulsen, *Antimicrobial susceptibility of Escherichia coli from community-acquired urinary tract infections in Europe: the ECO.SENS study revisited.* Int J Antimicrob Agents, 2012. **39**(1): p. 45-51 DOI: 10.1016/j.ijantimicag.2011.09.013.
29. Datsenko, K.A. and B.L. Wanner, *One-step inactivation of chromosomal genes in Escherichia coli K-12 using PCR products.* Proc Natl Acad Sci U S A, 2000. **97**(12): p. 6640-5 DOI: 10.1073/pnas.120163297.
30. Garcia-Fernandez, A., et al., *Characterization of plasmids harbouring qnrS1, qnrB2 and qnrB19 genes in Salmonella.* J Antimicrob Chemother, 2009. **63**(2): p. 274-81 DOI: 10.1093/jac/dkn470.
31. Carattoli, A., et al., *Identification of plasmids by PCR-based replicon typing.* J Microbiol Methods, 2005. **63**(3): p. 219-28 DOI: 10.1016/j.mimet.2005.03.018.
32. Wirth, T., et al., *Sex and virulence in Escherichia coli: an evolutionary perspective.* Mol Microbiol, 2006. **60**(5): p. 1136-51 DOI: 10.1111/j.1365-2958.2006.05172.x.
