# Supplementary figures and images for: Piggybacking on Niche Adaptation Improves the Maintenance of Multidrug‐Resistance Plasmids
Source: Mol Biol Evol. 2021 Mar 24;38(8):3188–201. doi: 10.1093/molbev/msab091 (PMC8321521; doi:10.1093/molbev/msab091)

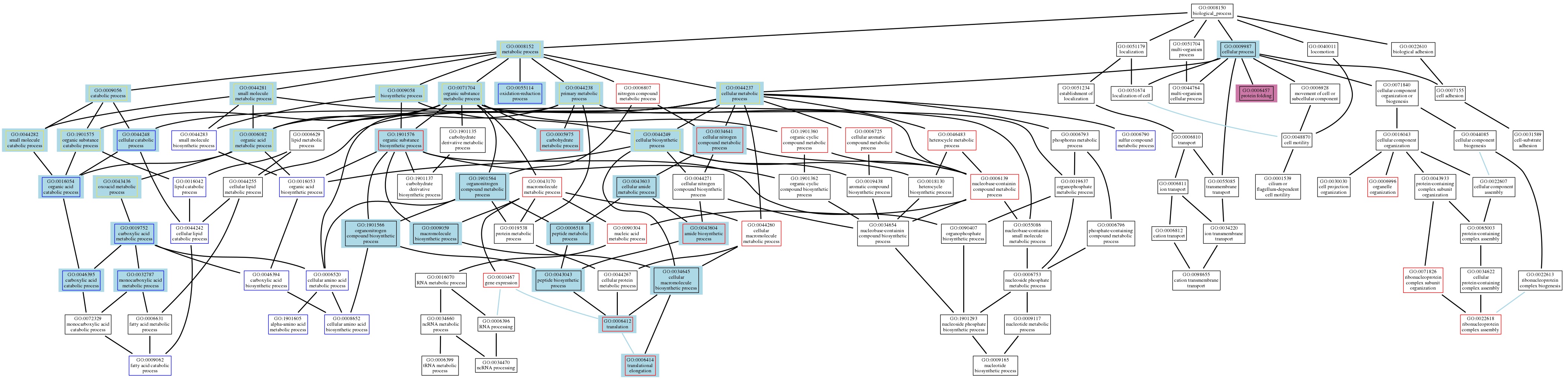

Supplement: msab091_Supplementary_Data [file msab091_supplementary_data.zip › Supplementary Figure 4.jpg]

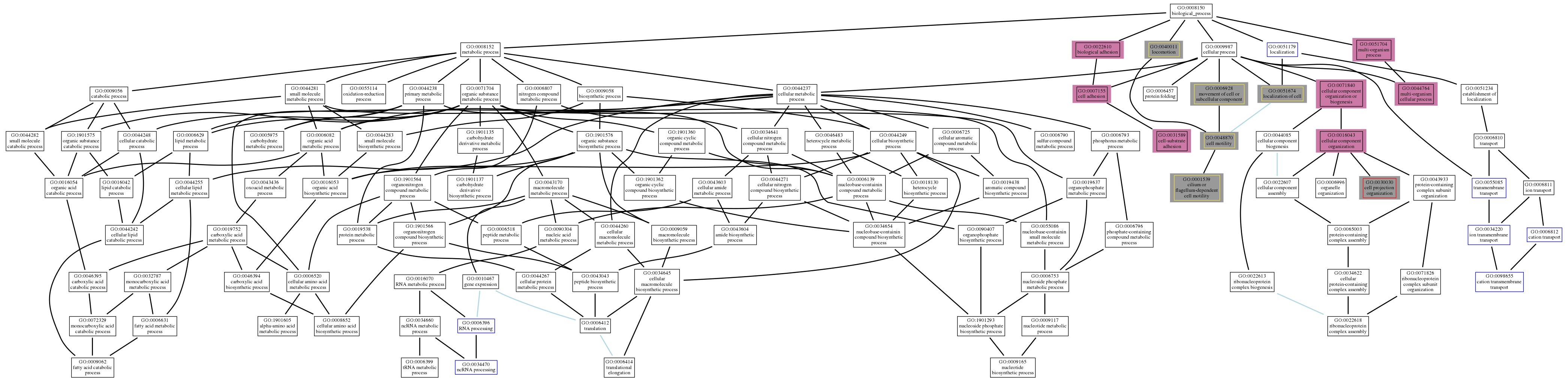

Supplement: msab091_Supplementary_Data [file msab091_supplementary_data.zip › Supplementary Figure 5.jpg]

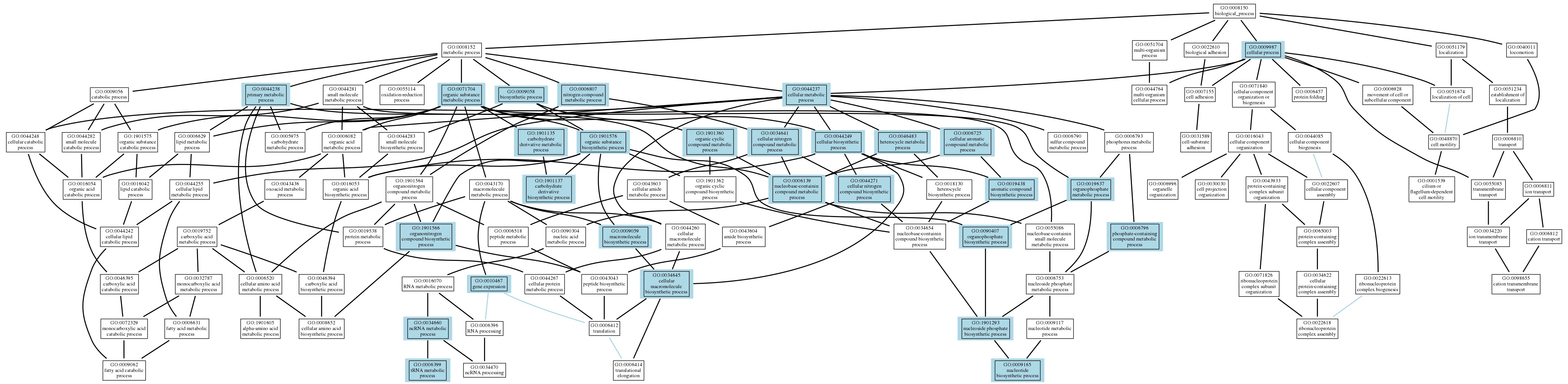

Supplement: msab091_Supplementary_Data [file msab091_supplementary_data.zip › Supplementary Figure 6.jpg]
